# Mitigating the outbreak of an infectious disease over its life cycle: A diffusion-based approach

**DOI:** 10.1371/journal.pone.0280429

**Published:** 2023-01-26

**Authors:** Xiaoming Li, Conghu Wang, Bin Jiang, Hua Mei

**Affiliations:** 1 Department of Business Administration, Tennessee State University, Nashville, Tennessee, United States of America; 2 Department of Public Administration, Renmin University of China, Beijing, China; 3 Department of Pharmacy Administration and Clinical Pharmacy, Peking University, Beijing, China; 4 Department of Chemistry & Physics, Belmont University, Nashville, Tennessee, United States of America; Education University of Hong Kong, CHINA

## Abstract

We first qualitatively divide the cycle of an infectious disease outbreak into five distinct stages by following the adoption categorization from the diffusion theory. Next, we apply a standard mechanistic model, the susceptible-infected-recovered model, to simulate a variety of transmission scenarios and to quantify the benefits of various countermeasures. In particular, we apply the specific values of the newly infected to quantitatively divide an outbreak cycle into stages. We therefore reveal diverging patterns of countermeasures in different stages. The stage is critical in determining the evolutionary characteristics of the diffusion process. Our results show that it is necessary to employ appropriate diverse strategies in different stages over the life cycle of an infectious disease outbreak. In the early stages, we need to focus on prevention, early detection, and strict countermeasure (e.g., isolation and lockdown) for controlling an epidemic. It is better safe (i.e., stricter countermeasures) than sorry (i.e., let the virus spread out). There are two reasons why we should implement responsive and strict countermeasures in the early stages. The countermeasures are very effective, and the earlier the more total infected reduction over the whole cycle. The economic and societal burden for implementing countermeasures is relatively small due to limited affected areas, and the earlier the less burden. Both reasons change to the opposite in the late stages. The strategic focuses in the late stages become more delicate and balanced for two reasons: the same countermeasures become much less effective, and the society bears a much heavier burden. Strict countermeasures may become unnecessary, and we need to think about how to live with the infectious disease.

## Introduction

There is currently a coronavirus (COVID-19) outbreak around the world that disrupts billions of people’s lives. Governments and organizations have to rely on various countermeasures: border closures, social distancing, the population quarantine, and the isolation of infected patients [1,2]. However, many responses are somewhat unorganized and quite different in various regions when facing similar diffusion patterns. A good example is how different countries treat early detection differently. Kim et al. [3] found that “The first confirmed COVID-19 case in South Korea was reported on Jan. 20, just a day before the first confirmed case in the U.S. was reported.” “By the time South Korea reported 204 confirmed cases on Feb. 21, the country had conducted a total of 16,400 tests. By the time the U.S. had 207 confirmed cases on March 4, it had performed 1,597 tests, 10 times fewer, according to the COVID Tracking Project.”

Epidemic models can simulate how infectious diseases spread and also show how our policies and actions alter the dynamics of an epidemic outbreak [1,2]. Analytical models are vital in supporting various decision-makings, including forecasting COVID-19 growth rates, modeling and forecasting the excess demand for products and services, and simulating government decisions (e.g., lockdown).

In addition, we have successfully applied key findings from biology into promoting and controlling new product and technology diffusions. For example, products and ideas may “grow through a person-to-person diffusion process analogous to the spread of an infectious disease” [4]. “The term "contagion" originated in biological science and is popularly used to signify the spread of disease through touch or other forms of close contact among individuals.” Using a social contagion lens, Angst et al. [5] theorized social contagion and built an important link between biological and social diffusions.

The opposite is also true. We need to employ key findings from the diffusion theory into the control on the current coronavirus outbreak and future ones. The key findings from the diffusion theory successfully prescribe relative speed and spatial patterns of diffusions over complex networks. Effective managerial and policy actions must be established on these speed and spatial patterns. Our contributions extend the theoretical diffusion knowledge on complex networks and consider the whole life cycle along multi-tier infectious chains with their disruptions. These results are valuable for leveraging the diffusion theory on controlling epidemics effectively and efficiently. We aim to answer the following three research questions: How to divide an infectious disease cycle into different stages? How do countermeasures in different stages cause different evolutionary dynamics? How to set up appropriate countermeasure strategies in different stages?

## Literature review

### The diffusion theory

**Fig 1:**
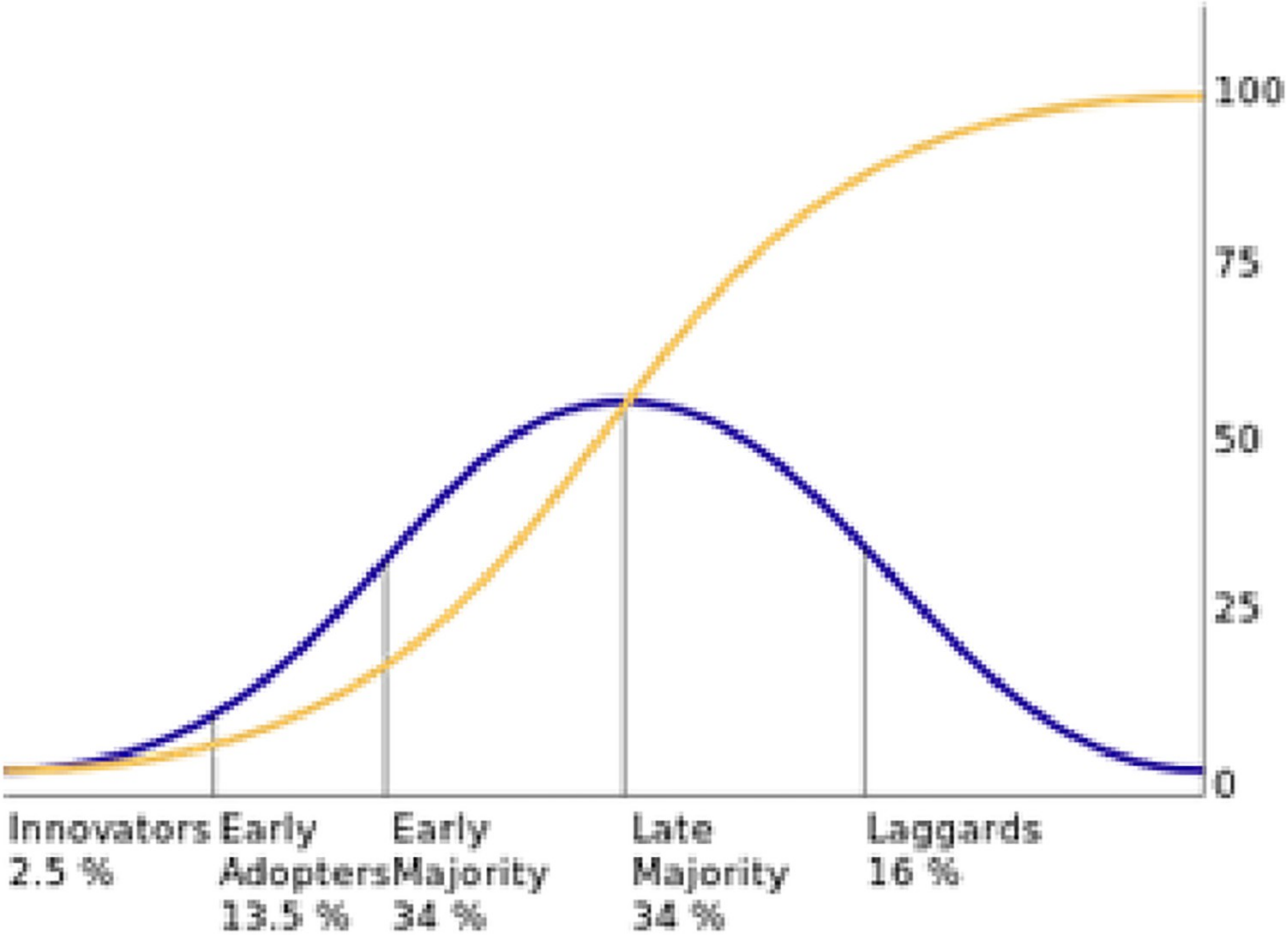
Adoption/Infection stages based on relative time and growth. (Robertsonm 1967, p. 16, Rogers 2003, p. 280).

The diffusion theory has become a vital part of management research and is widely applied in various fields, including management, policy, marketing, technology, and healthcare [6–10]. The theory successfully prescribes the relative speed and the spatial patterns of diffusions on complex networks. As shown in Fig 1, one key finding is that over time, the diffusion shows an S-shaped distribution with five distinct categories/stages: innovators, early adopters, early majority, late majority, and laggards [6,7,9–12]. “These categories correspond to extensive empirical evidence in quantitative terms” [10 p. 335]. “The method of adopter categorization just described is the most widely used in diffusion research today. It is essentially the only method of adopter categorization” [9 p. 282]. Terms in different categories such as “innovators” and “early adopters” are commonly used and well understood by the general public. “S-shaped adopter distributions closely approach normality” as in Fig 1 [9 p. 280, 11 p. 16].

We have two key takeaways from the above categorization method. First, we must adopt a holistic view over the life cycle of diffusion. When we implement countermeasures, we need to consider their impact on that particular time/stage as well as on the whole life cycle to the very end of an epidemic. Second, different categories/stages tend to demonstrate their own characteristics. The diffusion theory literature has summarized three major traits: socioeconomic status, personality traits, and communication behavior. The diffusion of an infectious disease shows many similarities. When we implement countermeasures, we need to adopt distinct strategic focuses for different stages.

### Epidemiologic models

Mathematical models are vital for fighting epidemics. [1,2,13–15]. Siettos and Russo [15] provided a comprehensive review of three main approaches for the surveillance and modeling of infectious disease dynamics: (1) statistical methods for surveillance of outbreaks and identification of spatial patterns in real epidemics, (2) mathematical models within the context of dynamical systems used to forecast the evolution of a “hypothetical” or on-going epidemic spread, and (3) machine learning/expert methods for the forecasting of the evolution of an ongoing epidemic. Also, importantly, Siettos and Russo [15] presented basic relevant concepts underpinning their implementation and practice with representative works. The celebrated Kermack and McKendrick [16] model (i.e., the susceptible-infected-recovered (SIR) model) is the basis of mathematical/mechanistic state-space models. Mechanistic models simulate the ways how infectious diseases spread and can fit many future transmission scenarios by adjusting assumptions and model parameters [13,14]. Mechanistic models can make long-term predictions. Because epidemiologic data are evolving and we face a lot of uncertainties and dissimilar reactions, we need to carry out sensitivity analyses to figure out the converging patterns.

Controlling an epidemic appears to be one major challenge nowadays. Researchers have made improvements from various perspectives. Reppas et al. [17] applied a novel equation-free computational framework for multi-scale analysis to effectively analyze the macroscopic emergent behavior of complex epidemic models. They thus solve a critical issue that bridges the diverse space and time scales from the microscopic scale, including the immune mechanisms, host-microbe, and host-host interactions, to the macroscopic population scale with an emergent epidemic. Siettos [18] exploited the equation-free approach and optimization methods to bridge detailed individual-based epidemic models with coarse-grained, system-level analysis within a pair-wise representation perspective. Siettos [18] could therefore perform steady state computations, stability computations, and continuation and numerical bifurcation analysis of the complex-emergent dynamics while bypassing the need of analytical derivation of closures for the macroscopic-level equations.

Some researchers investigate the important role of asymptomatic infections in an epidemic. Russo et al. [19] provided prompt estimates for the day-zero of the COVID-19 outbreak in Lombardy, Italy; the actual number of asymptomatic infected cases in the total population until March 8, 2020; and the basic and the effective reproduction numbers. Russo et al. [19] model two compartments of infected individuals: (1) asymptomatic or experiencing very mild symptoms and (2) mild to severe symptoms. Note that COVID-19 was in the early stages from January to March 2020 in Italy. “As mentioned in the methodology, within the first period of the outbreak the number of confirmed cases was approximately equal to the number of hospitalized cases, i.e. there was no sampling strategy. Furthermore, as also reported for the later period, tests were conducted only for those who seeked medical care and had symptoms like fever and coughing. Thus, people who did not seek for medical attention were tested very scarcely” [19]. Most asymptomatic cases were not reported in the early stages of COVID-19. Russo et al. [19] successfully bridged the gap between asymptomatic and symptomatic cases and thus provide more accurate estimates for the effective control policies in real time. Russell et al. [20] accommodated asymptomatic cases to adjust reported cases such that they can correctly investigate how internationally imported cases cause an internal spread of COVID-19, due to different international travel restrictions. Liu et al. [21] incorporated asymptomatic and symptomatic, reported and unreported data to model the COVID-19 transmission dynamics in China, South Korea, Italy, France, Germany, and the United Kingdom. Liu et al. [21] evaluated both the early exponential growth phase before the government implementation of major control measures and the next phase when these measures result in a time-dependent exponentially decreasing number of cases. Brown [22] presented a customized susceptible, exposed, infected, and recovered compartmental model for illustrating the asymptomatic spread of COVID-19 at Boston University. Brown [22] found that the spread of COVID-19 is related to the exogenous infection rate in the community and is reduced by more frequent testing, effective contact tracing, and vaccination.

### A diffusion-based approach

Our approach is to integrate the diffusion theory with a standard mechanistic model. The approach has two steps: qualitative first and then quantitative. Qualitatively, the mechanics in the spread pattern of an infectious disease is the same as in the diffusion theory for innovation. We qualitatively divide a cycle of an infectious disease outbreak into five distinct stages by following the adoption categorization from the diffusion theory. Next, we apply a popular mechanistic model, the SIR model, to simulate a variety of transmission scenarios and to quantify the benefits of various countermeasures. In particular, we quantitatively divide an outbreak cycle into five stages by introducing a newly infected variable and following exact numeric percentages from the adoption categorization in the diffusion theory. We therefore reveal diverging patterns of countermeasures in different stages.

### Infection stages in a disease diffusion

At the beginning, the whole population is basically susceptible to a new infectious disease. Then, more and more people get infected and recovered, so the susceptible compartment has fewer and fewer people. The growth of an infectious disease typically follows a normal curve [12]. These normal-like curves will be altered (e.g., flattening the curve, second wave, third wave, etc.) when we change transmission and recovery processes through lockdown, reopening business, vaccination, etc. This paper focuses on how to depress the curve more effectively.

Following the same rationale as in the diffusion theory and the Bass Model, we now qualitatively divide the whole cycle of a disease outbreak sequentially into five stages below:

Introduction–people infect the disease the first time in humans. The further diffusion occurred in a small area between humans in droplets from coughing and sneezing and touching/shaking hands.

Early infectors–they are family members and doctors and nurses of newly infected. They have close contacts with newly infected that first develop symptoms. They tend to catch the virus by surprise because they are not aware of the infected disease. The spread channel remains the same by droplets from coughing and sneezing and touching/shaking hands. When there are enough early infectors, the critical mass [9 p. 343] occurs after this tipping point at which enough infectors make the infection diffusion self-sustaining.

Early majority–people obtain the infection through major societal hubs: buses, subways, trains, airports and airplanes, and other confined social gatherings like concerts. Modern societal hubs may push new infected cases to an upward outburst; we may never see such a rate before.

Late majority–people are infected in the further tier of the infectious chain and in further areas. Infectiousness becomes more stable. New cases have peaked and start to decrease.

Laggards–people are infected in the furthest tier of the infectious chain and in furthest areas. At this stage, the virus becomes less infectious and people are more prepared. So, the infection rate decreases much further.

### Qualitative focuses and their implementations

To stop and mitigate the widespread of infectious diseases, governments and organizations need to work together to take a holistic approach on all infection stages over the entire life cycle, based on the above qualitative characteristics in each stage. Strategic focuses and their implementations need to adapt to specific characteristics of each infection stage. The key is to closely chase both speed and spatial diffusions over the entire cycle, to cut the disease spread at critical times and locations.

At the earliest stage of mutators, the focus is on prevention. The strategic measures are preventative to maintain a clean environment. It would be ideal if we can prevent people from getting the virus at the first place. The control effort is the easiest among all stages, due to the least infected people within the smallest area.

At the stage of early infectors, the strategic focus is on establishing early detection systems around mutators’ family members and healthcare facilities and hospitals. Essential measures include appropriate training on doctors and nurses; sufficient medical equipment, supplies, and medicines; and also importantly early detection systems. Doctors and nurses should be able to diagnose and treat patients quickly, and quarantine/isolate both confirmed and suspected patients and those affected. The best solution is to cut the infection at its source. It is a race against time to avoid the infectious diffusion reaching the critical mass point. It is always better safe than sorry in these two early stages, because costs and efforts become exponentially large in later stages. The most important factor is time while the system remains responsive, flexible, and agile.

When comes to the early majority stage, the outbreak reaches major societal hubs: buses, subways, trains, airports and airplanes, and other confined social events. Strategic focuses become broader, which tends to require more communications and collaboration among higher-level governments and health organizations. It now becomes required to establish nationwide warning systems, monitor and control affected transportation, and regulate confined societal events. It is still feasible to lock down these major social hubs, sometimes major cities, to overcome the rapid diffusion of the disease.

At the late majority stage, people are now infected in the further tier of the infectious chain and in further areas. Infected people are further scattered in much bigger areas. The disease management is a joint effort of all involved. Strategic focuses become delicate and balanced over broader areas because each decision affects a lot of people, and each decision needs all parties involved. We now have additional clear data from early stages: virus data, genome sequence, location, time, infected people, diffusion pattern, big data mappings, etc. Efficiency becomes key due to much better diagnostic and therapeutic approaches. The decision should be based on data with careful deliberation on a cost-benefit analysis: cost of a strategic change vs. its benefit in such a broad area.

At the last laggards stage, people are infected in the furthest tier of the infectious chain and in furthest areas. At this stage, the virus becomes less infectious (e.g., herd immunity) and people are more prepared. The strategic focus shifts to proficiently eradicate the infectious disease (e.g., smallpox) or live with it (e.g., COVID-19 becomes an endemic like flu). Countermeasures like lockdown and business closures become meaningless due to the highest societal and economic cost and the least remaining susceptible population.

Overall, a first-responsive-then-efficient strategy is appropriate to mitigate such epidemics.

## Method

### An SIR model for quantifying dynamics

We use a popular mathematical model, the SIR model, to simulate various dynamics of an epidemic. Our method does not involve human participants. The SIR model was first used by Kermack and McKendrick [16] and has subsequently been widely applied to a variety of diseases, especially airborne diseases [23]. *S*_*t*_, *I*_*t*_, and *R*_*t*_ denote the number of susceptible, infected, and recovered individuals in the population at time *t*, respectively. *β* is the transmission rate constant and *γ* is the recovery rate constant. *N* = *S*+*I*+*R* is the total population, which is a constant.

The SIR model equations include

$$\frac{dS}{dt}=-\frac{\beta }{N}S_{t}I_{t},$$ (1)

$$\frac{dI}{dt}=\frac{\beta }{N}S_{t}I_{t}-{\gamma I}_{t},$$ (2)

$$\frac{dR}{dt}={\gamma I}_{t}.$$ (3)

These three equations show how the whole population progresses from susceptible to infected to recovered. Eq 1 is the infection rate from susceptible to infected, $-\frac{\beta }{N}S_{t}I_{t}$, where the negative sign means that the susceptible is decreasing over time. Eq 3 shows the recovery from infected to recovered, *γI*_*t*_, where the recovered compartment is increasing over time. Recovered individuals remain immune permanently. Eq 2 is the change rate of the infected compartment, $\frac{\beta }{N}S_{t}I_{t}-{\gamma I}_{t}$, transited from susceptible $\frac{\beta }{N}S_{t}I_{t}$ minus leaving to recovered *γI*_*t*_.

$R_{0}=\frac{\beta }{\gamma }$ is the basic reproduction number because the susceptible individuals at the beginning are basically the whole population. The effective reproduction is $R_{e}=\frac{S_{t}}{N}\frac{\beta }{\gamma }$ for different susceptible individuals at different times.

We therefore have two important managerial questions:

1. Will an infectious disease spread further?
2. How many individuals will catch the infectious disease over the whole cycle?

For decision-making, we aim to stop the spread of infectious disease and to lower total people infected over the whole cycle. When R_*e*_>1, the infectious disease will spread further; when R_*e*_≤1, the infectious disease will decrease monotonically to 0. With a starting $R_{0}=\frac{\beta }{\gamma }>1$, we need to have $\frac{S_{t}}{N}\leq \frac{1}{R_{0}}$ (or equivalently $\frac{R_{t}}{N}\geq 1-\frac{1}{R_{0}}$) such that R_*e*_≤1; in words, the recovered group must be greater than $1-\frac{1}{R_{0}}$ in order to stop the spread.

### Dividing stages in an outbreak cycle

To show how useful countermeasures change the progression of infectious diseases in different stages, now we use the following discrete version of the SIR model:

$$S_{t+1}-S_{t}=-\frac{\beta }{N}S_{t}I_{t}$$

$$I_{t+1}-I_{t}=\frac{\beta }{N}S_{t}I_{t}-{\gamma I}_{t}$$

$$R_{t+1}-R_{t}={\gamma I}_{t}$$

Also, infectious disease data are collected and posted periodically, for example, every day.

Following the diffusion theory and the Bass Model, we are interested in newly infected cases. This is also consistent with governments’ data collections and postings–new confirmed cases. Also importantly, the cumulative new infected fractions are always between 0% and 100%, which makes our comparisons with different dynamics consistent and easier. On the other hand, the cumulative infected (i.e., sum of *I*_*t*_) can change considerably due to different recovery rates.

We define the new infected in period *t*+1 as $I_{t+1}^{\prime }=\frac{\beta }{N}S_{t}I_{t}$, which is how many individuals transmitted from susceptible to infected in period *t*+1.

From SIR Model, we have $I_{t+1}-I_{t}=\frac{\beta }{N}S_{t}I_{t}-{\gamma I}_{t}$, so $I_{t+1}-I_{t}=I_{t}^{\prime }-{\gamma I}_{t}$. We also have

$I_{t}-I_{t-1}=\frac{\beta }{N}S_{t-1}I_{t-1}-{\gamma I}_{t-1}$ and $I_{t+1}-I_{t}=\frac{\beta }{N}S_{t}I_{t}-{\gamma I}_{t}$, Therefore, we know

$\sum_{t=0}^{End-1}\left(I_{t+1}-I_{t}\right)=\sum_{0}^{End-1}\left(\frac{\beta }{N}S_{t}I_{t}-{\gamma I}_{t}\right)$ and $I_{End}-I_{0}=\sum_{t=0}^{End-1}I_{t+1}^{\prime }-\sum_{t=0}^{End-1}{\gamma I}_{t}$. So $\sum_{t=0}^{End-1}I_{t+1}^{\prime }=\sum_{t=0}^{End-1}{\gamma I}_{t}=\gamma \sum_{t=0}^{End-1}I_{t}$ when initially *I*_0_≈0 is very small and at the end *I*_*End*_ = 0. In words, over the whole cycle, the sum of infected is the product of the average recovery periods (*γ*^−1^) and the sum of new infected: $\sum_{t=0}^{End-1}I_{t}=\frac{1}{\gamma }\sum_{t=0}^{End-1}I_{t+1}^{\prime }.$ The difference between $I_{t}^{\prime }$ and *I*_*t*_ is due to the fact that one particular individual can only be newly infected once in a period as in $I_{t}^{\prime }$ but one particular individual can remain infected in multiple periods (i.e., a recovery takes multiple periods) as in *I*_*t*_.

Without loss of generality, we assume *N* = 1. Then, the set of susceptible, infected, and recovered variables represents the fraction of the total population in each of the three compartments. Define the cumulative new infected till period *t* as $F\prime (t)=I_{0}+\sum_{x=1}^{t}I_{x}^{\prime }$, where *I*_0_ is the initial infected at the beginning of an infectious disease cycle, where *t* = 1,2,3,… End and *x* is a positive integer till *t*. Then, the total fraction of the population getting infected over the whole cycle is *F*′(End) = *R*_End_.

Define the inverse function of cumulative new infected, *Q*(*p*), as the minimum period such that $F\prime (t)\geq p:{Q(p)=F\prime }^{-1}(p)=min\;\{t:F\prime (t)\geq p\}$. In this way, we can divide the whole cycle into 5 stages: introduction 0≤*t*<*Q*(0.025), early infectors *Q*(0.025)≤*t*<*Q*(0.16), early majority *Q*(0.16)≤*t*<*Q*(0.5), late majority *Q*(0.5)≤*t*<*Q*(0.84), and laggards *Q*(0.84)≤*t*≤*Q*(1). These periods are corresponding to proportional cumulative new infected in each category as specified in the diffusion theory: 0.025, 0.135, 0.34, 0.34, and 0.16, respectively.

We argue that the new infected $I_{t}^{\prime }$ and its cumulative function are better measures for investigating dynamics over the whole cycle of an infectious disease. Also, the cumulative new infected is always between 0 and 1, which provides a consistent measure for all possible cases. Below we show how to use above concepts in a variety of settings and how we can draw consistent managerial insights.

### Quantifying dynamics in different stages

SIR data for COVID-19 are still evolving, and many countries have quite different empirical data [23]. So, we use a wide range of SIR parameters to answer our research questions. Using empirical data from three US states (California, New York, and Indiana), Bertozzi et al. [23] show that *γ* ranges from 0.06 to 0.19 and R_0_ from 2.1 to 4.4. Lin et al. [24] summarize a mean infectious period (i.e., *γ*^−1^) as 5 days for COVID-19.

Thus, we use *γ* = 0.125 and R_0_ = 3 (or equivalently *β* = 0.375) as our base model. (We conduct a sensitivity analysis by changing these parameters in the next section.) We set the following initial values in period 0: *S*_0_ = 0.999999, *I*_0_ = 0.000001, and *R*_0_ = 0. To accommodate a variety of parameters in our sensitivity analysis, we end each simulation run in period 2000.

The susceptible fraction is reducing over time while the recovered fraction is increasing over time. The infected fraction tends to peak in the middle of the cycle. All changes are slow at the beginning, then fast in the middle, and finally slow again at the end.

Comparing *I*_*t*_ with $I_{t}^{\prime }$, the changes of new infected are following those of infected. The cumulative new infected at the end is also the recovered fraction of the population, which is always between 0% and 100%. The cumulative infected can be greater than 1 because the infected may take multiple periods to recover. They both share similar growth patterns.

To show dynamics after countermeasures for controlling the outbreak of an infectious disease, we classify countermeasures into three categories: quicker recovery rate, lower transmission rate, and both, respectively. A quicker recovery rate typically is caused by better healthcare, including prompt detection and diagnosis, better therapeutics, improved medicine, etc. A lower transmission rate can be attributed to increased personal hygiene, personal protective equipment, social distance, school and event closing, lockdown, contact tracing, and quarantine. Multiple countermeasures simultaneously can produce both a quicker recovery rate and a lower transmission rate.

We introduce three changes into our base model

(1) the recovery rate *γ* is doubled from 0.125 to 0.25;
(2) the transmission rate *β* is reduced by half from 0.375 to 0.1875;
and (3) we simultaneously double the recovery rate *γ* and reduce the transmission rate *β* by half: *γ* increases from 0.125 to 0.25 and *β* decreases from 0.375 to 0.1875.

To show these changes over the whole cycle, we implement the above changes in five stages: introduction, early infectors, early majority, late majority, and laggards, respectively. Because the cumulative new infected in the introduction stage is 0.025, we implement the changes in period *Q*(0.0125), i.e., the half-point $\frac{0.025}{2}$. In the same manner, we implement the changes in period *Q*(0.0925), *Q*(0.33), *Q*(0.67), and *Q*(0.92), for early infectors, early majority, late majority, and laggards, respectively. We only implement one parameter change over the whole cycle. Note $0.0925=0.025+\frac{0.135}{2},0.33=0.025+0.135+\frac{0.34}{2},0.67=0.025+0.135+0.34+\frac{0.34}{2}$, and $0.92=0.025++0.135+0.34+0.34+\frac{0.16}{2}$.

In some cases, not 100% of people will get infected at the end, just like incomplete adoption in the diffusion theory (Rogers, 2003, p. 281). This means these late stages are merged together.

For our base model where R_0_ = 3 (or equivalently *β* = 0.375) and *γ* = 0.125, *Q*(0.0125) = 41, *Q*(0.0925) = 50, *Q*(0.33) = 57, *Q*(0.67) = 64, and *Q*(0.92) = 80, respectively. Therefore, to represent countermeasures in introduction, early infectors, early majority, late majority, and laggards, we change recovery rate and transmission rate accordingly in periods 41, 50, 57, 64, and 80, respectively.

**Fig 2:**
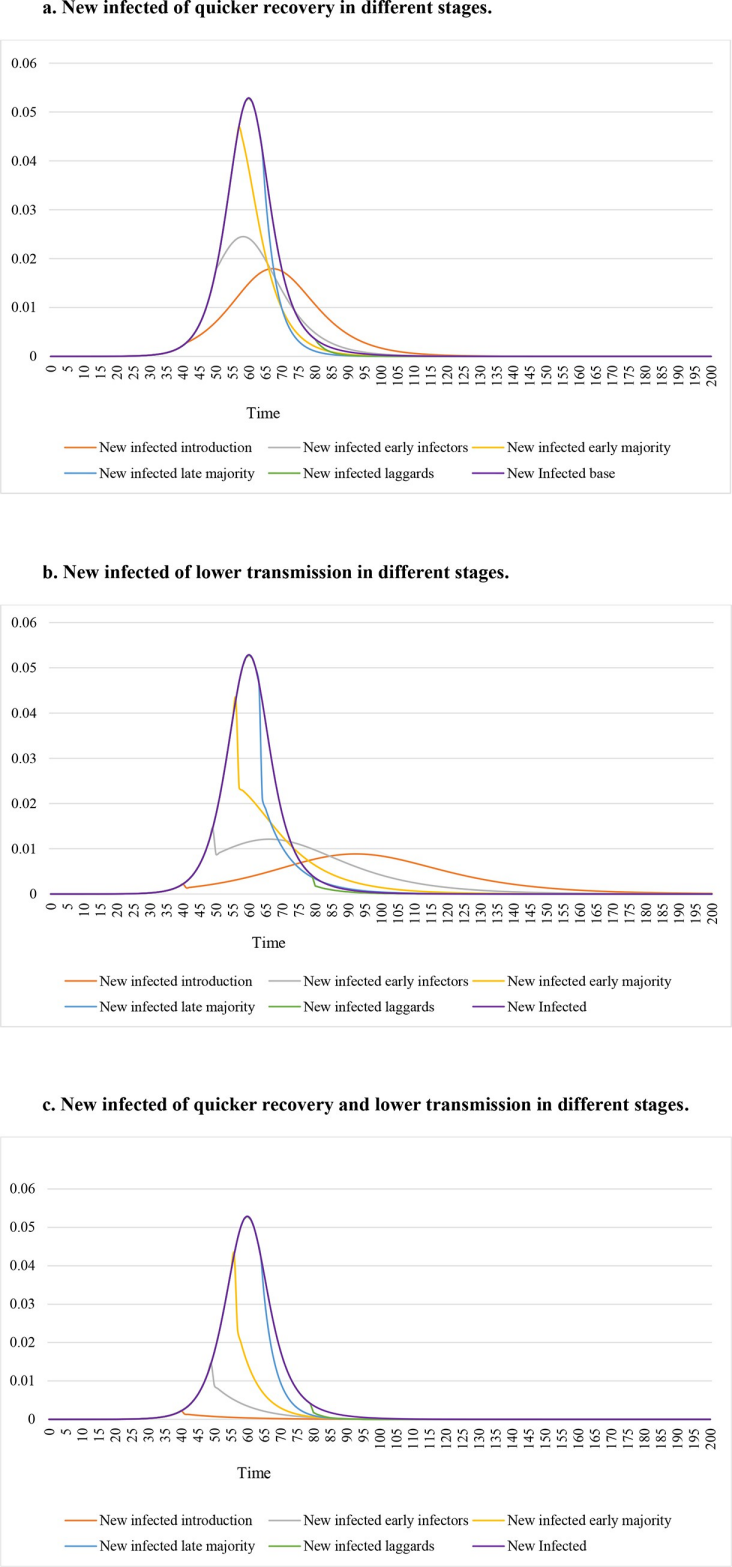
a. New infected of quicker recovery in different stages. b. New infected of lower transmission in different stages. c. New infected of quicker recovery and lower transmission in different stages.

Fig 2A shows new infected over the whole cycle with quicker recovery in introduction, early infectors, early majority, late majority, and laggards stages, respectively. The orange line doubles the recovery rate in the introduction stage, and is pushed to the right furthest and thus has the lowest peak. The gray, gold, blue, and green lines represent a quicker recovery change in early infectors stage, early majority, late majority, and laggards stages, respectively. The purple line is our base model with *γ* = 0.125 and *R*_0_ = 3, which has the highest peak. If we implement quicker recovery earlier, new infected are depressed earlier and also are lowered in peak.

Fig 2B shows the same pattern for a lower transmission rate introduced in introduction, early infectors, early majority, late majority, and laggards stages, respectively. When *β* is halved, the line has a sudden drop. Fig 2C shows exactly the same pattern for a higher recovery rate and a lower transmission rate introduced in introduction, early infectors, early majority, late majority, and laggards stages, respectively. The differences to the base model are the biggest in Fig 2C.

**Fig 3:**
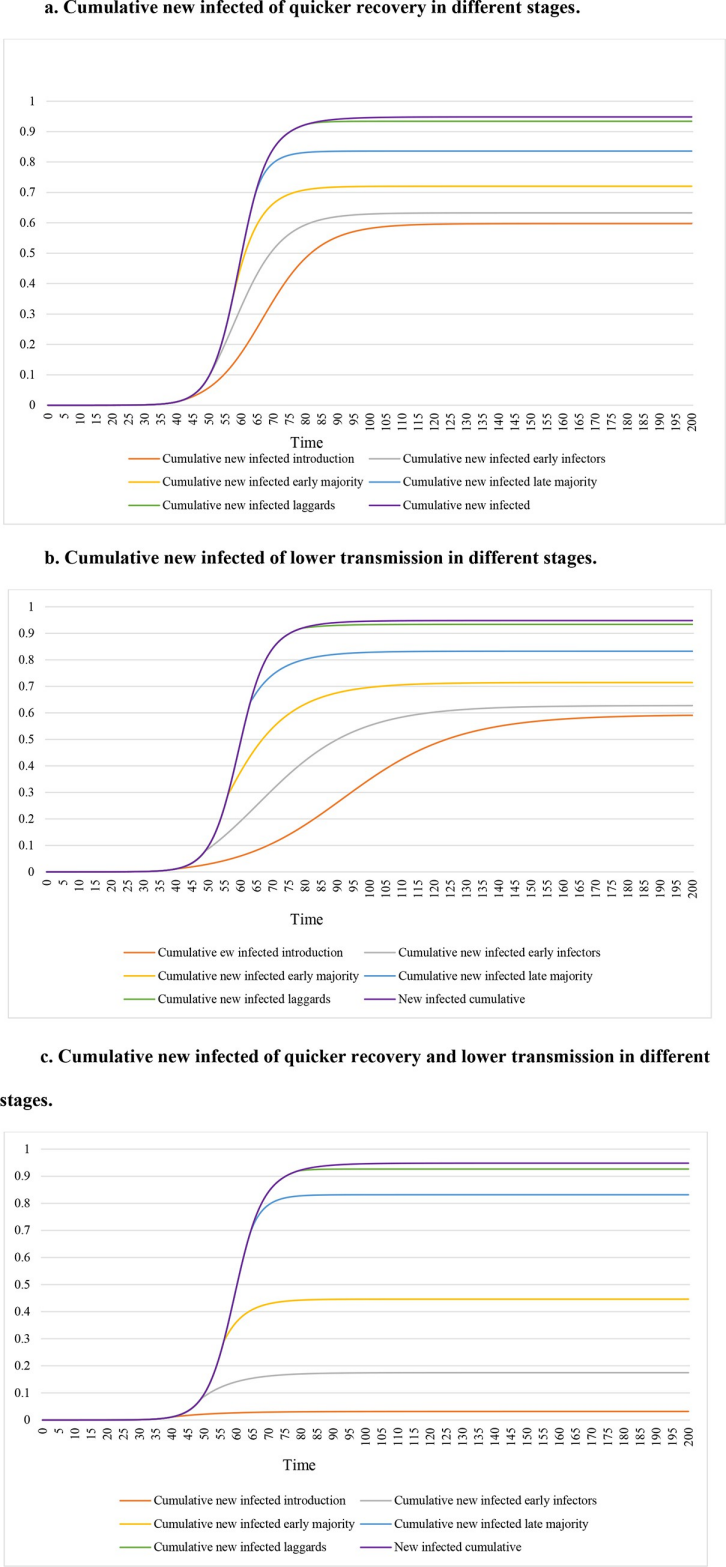
a. Cumulative new infected of quicker recovery in different stages. b. Cumulative new infected of lower transmission in different stages. c. Cumulative new infected of quicker recovery and lower transmission in different stages.

Fig 3A, 3B and 3C show how the cumulative new infected change over the cycle. It is more clear to see the monotonical relationship among when changes are implemented in different stages. Countermeasures implemented in the introduction stage always bring the largest benefits, following by early infectors, early majority, late majority, and laggards stages. When to implement countermeasures can be more important than which countermeasures themselves. For example, implementing quicker recovery or lower transmission countermeasures in early stages (introduction, early infectors, and early majority) results that the cumulative new infected at the end is less than 80%, but implementing both quicker recovery and lower transmission countermeasures in late stages (late majority and laggards) results that the cumulative new infected at the end is greater than 80%.

### Sensitivity analysis

We let *γ* = 0.0625, 0.125, or 0.25, and R_0_ = 1.5, 3, or 6, respectively. We focus on the total infected fraction of the population. Therefore, we have 9 cases as shown in Table 1.

**Table 1:** Values of *β*, *γ*, and *R*_0_ in nine cases.

Case No.	*β*	*γ*	R_0_
1	0.09325	0.0625	1.5
2	0.1875	0.0625	3
3	0.375	0.0625	6
4	0.1875	0.125	1.5
5	0.375	0.125	3
6	0.75	0.125	6
7	0.375	0.25	1.5
8	0.75	0.25	3
9	1.5	0.25	6

**Fig 4:**
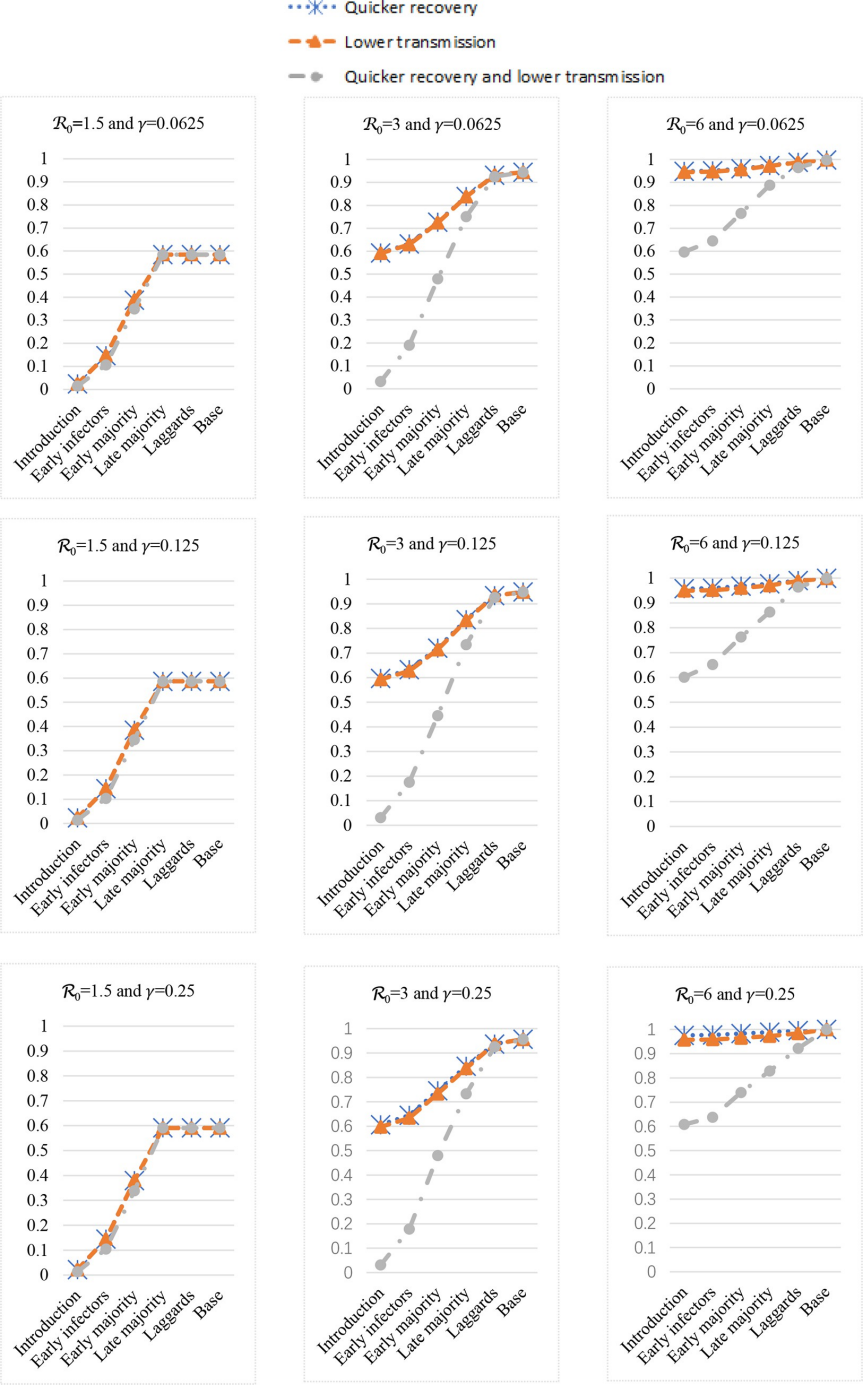
The cumulative infected (i.e., the recovered fractions or total infected) with different countermeasures.

Fig 4 shows the cumulative new infected (i.e., the total infected fraction or the total recovered fraction) at the end of a cycle. The first column shows 3 cases where R_0_ = 1.5, the second column shows 3 cases where R_0_ = 3, and the third column shows 3 cases where R_0_ = 6. The first row shows 3 cases where *γ* = 0.0625, the second row shows 3 cases where *γ* = 0.125, and the third row shows 3 cases where *γ* = 0.25.

Among the three columns, the figure shapes are quite different. So, R_0_ is very important. The cumulative new infected is increasing in R_0_. The countermeasures benefits are larger if the starting implementation period of such a change is earlier. When R_0_ = 1.5, roughly 40% of the population will never be infected even without any countermeasure; more than 90% can stay non-infected if we implement countermeasures in the introduction stage; countermeasures’ benefits are decreasing if we implement such countermeasures in the later stages. When R_0_ = 3, less than 10% can stay non-infected if we implement countermeasures at the laggards stage or we do not implement any change; but, more than 90% can stay non-infected if we act in the introduction stage. So, which stage to act is critical to the ending result of controlling an epidemic. When R_0_ = 6, again we have to act promptly; cumulative infected become more than 90% in a very short number of periods.

Regarding countermeasures, the benefits of a quicker recovery rate are similar to those of a lower transmission rate. Obviously, it is always the best to have the benefits of both a quicker recovery rate and a lower transmission rate.

Among the three rows, i.e., same reproduction numbers but different recovery rates, the figure shapes are essentially the same. So, the recovery rate is trivial for the same R_0_. The countermeasures benefits are larger if the starting implementation period of such a change is earlier. Promptness is very important.

We then further run small changes of the transmission rates to reflect different levels of countermeasures, e.g., lockdown, business and school closing, partial opening, etc. We use our base model with *γ* = 0.125 and R_0_ = 3 (or *β* = 0.375) as the starting point. We lower the transmission rate to 0.35, 0.3, 0.25, 0.2, 0.15, 0.1, and 0.05, respectively. We have the same finding as before: we will have a more total infected reduction if we implement countermeasures earlier. In addition, stricter countermeasures will have a larger impact on the total infected reduction. The relationship is monotonic. If we implement countermeasures too late, the total infected over the whole cycle is almost the same as in our base model, with not much total infected reduction. There is the upper bound for the cumulative number of the new infected variable, 94.83% in our base model. Our lowest total infected is 1.60%, caused by the lowest *β* = 0.05 in the introduction stage. 1.60% is the lower bound for the cumulative number of the new infected variable in these scenarios.

### Reopening businesses

Another type of decision-making is when to reopen businesses. We will have a higher transmission rate once we restart our economic and social activities. Also, more infected people may cause more congestions at healthcare facilities, which in turn may cause a slower recovery rate. We make three parameter adjustments to reflect changes of reopening businesses: (1) the recovery rate *γ* is halved, (2) the transmission rate *β* is doubled, and (3) we simultaneously halve the recovery rate *γ* and double the transmission rate *β*, respectively. Our base models are the same as the nine cases in Table 1. Again we implement the above three parameter changes in the introduction, early infectors, early majority, late majority, and laggards stages, respectively.

**Fig 5:**
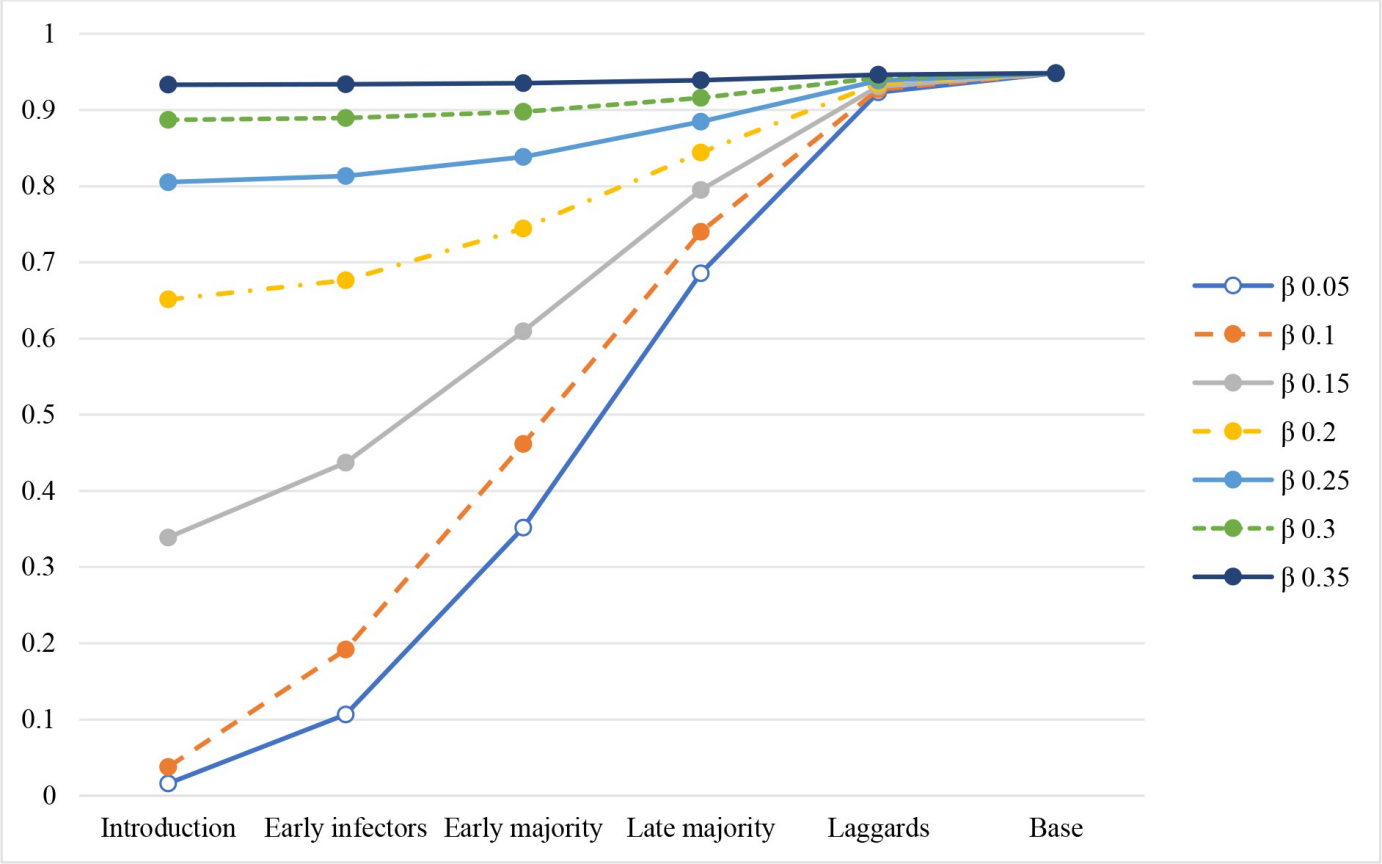
The cumulative infected (i.e., the recovered fractions or total infected) with different countermeasures on transmission rates (*β* beta).

The infected fraction changes are exactly the opposite of those of countermeasures in the previous section. Fig 5 shows detailed changes. If we reopen businesses earlier, we will have a higher infected fraction. When R_0_ = 1.5, roughly 40% of the population will never be infected in our base model. So, the late majority and the laggards stages are merged together as in three cases in the left column. We can find that the total infected increase dramatically from near 60% to near 95% if we reopen businesses in the early stages. When R_0_ = 3 and R_0_ = 6, the infected fraction in our base model is already more than 90%. So, reopening businesses means that the infected fraction will be higher and approaching 100%.

Among the three rows, i.e., same reproduction numbers but different recovery rates, the results are essentially the same. We now run more simulations with small transmission rate changes. We use our base model with *γ* = 0.125 and R_0_ = 3 (or *β* = 0.375) as the starting point. We increase the transmission rate to 0.4, 0.45, 0.5, 0.55, 0.65, 0.7, and 0.75, respectively.

**Fig 6:**
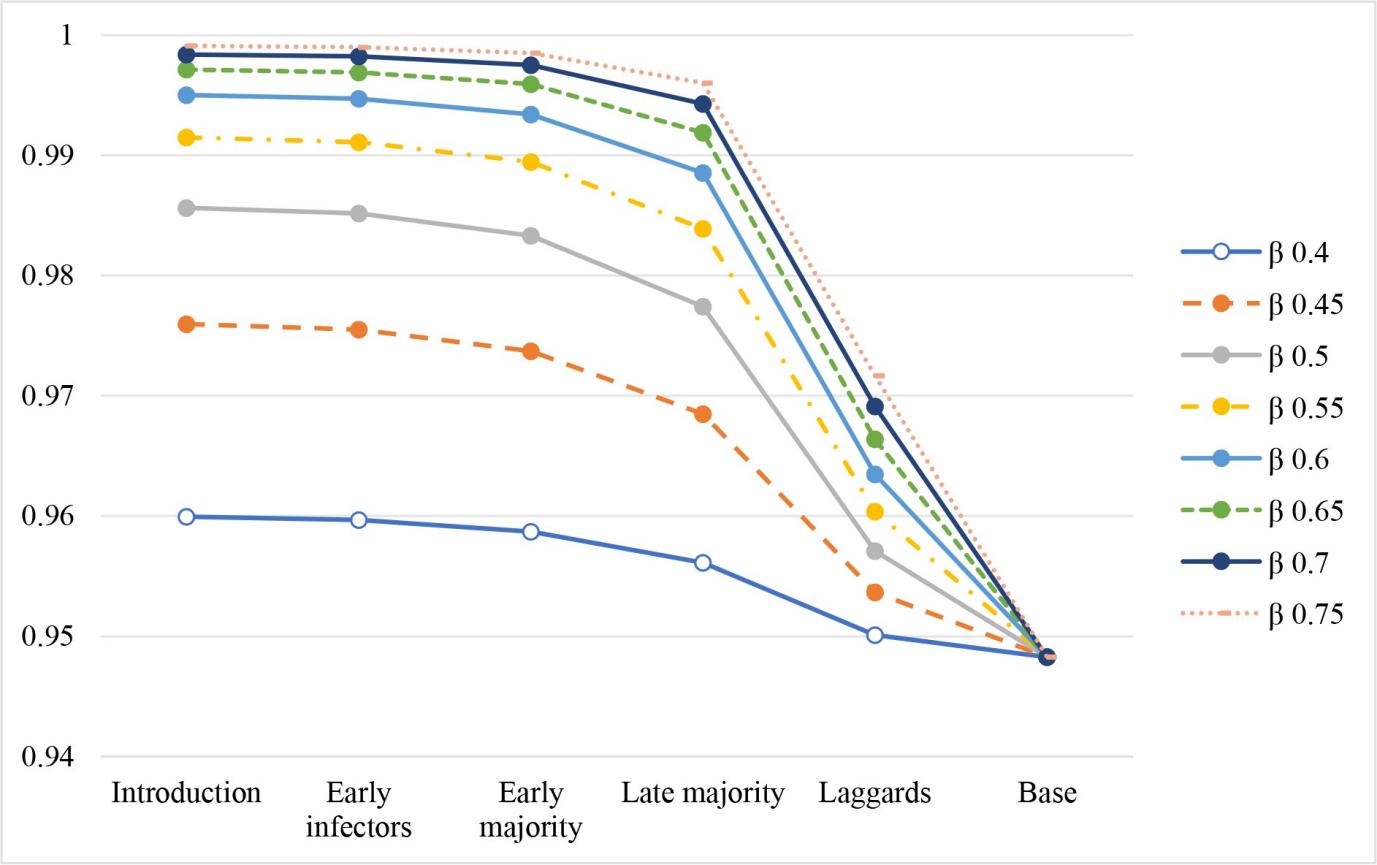
The cumulative infected (i.e., the recovered fractions or total infected) with different levels of reopening businesses.

**Fig 7:**
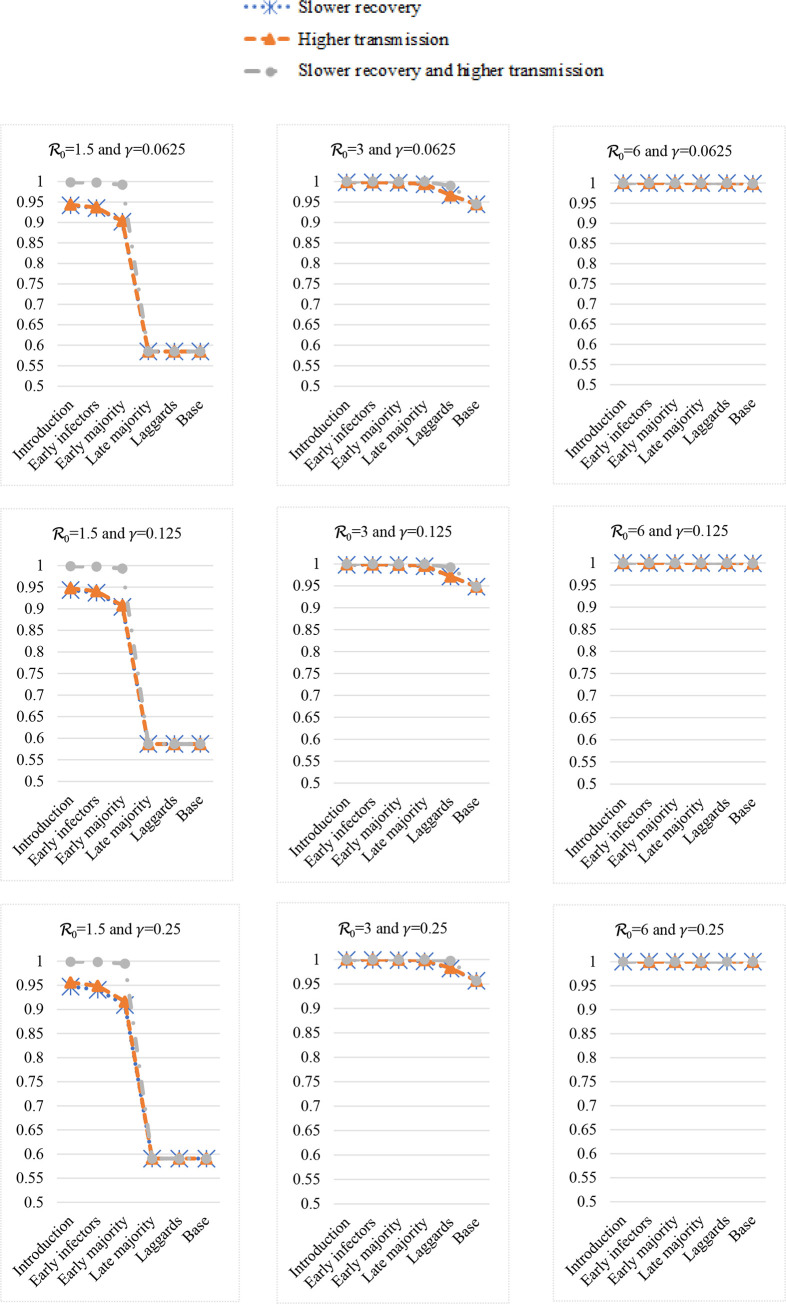
The cumulative infected (i.e., the recovered fractions or total infected) with reopening businesses.

We have the same finding as before, as shown in Fig 6: we will have a higher total infected if we reopen businesses earlier. In addition, a full opening (i.e., a higher transmission rate) will cause a higher total infected than a partial opening (i.e., a lower transmission rate). The relationship is monotonic. If we do not reopen businesses until very late, the total infected over the whole cycle is almost the same as in our base model, not much total infected increase. There is the lower bound for the cumulative number of the new infected variable, 94.83% in our base model. Our highest total infected is 99.91%, caused by the highest *β* = 0.75 in the introduction stage. 99.91% is the upper bound for the cumulative number of the new infected variable in these scenarios (Fig 7).

## Conclusions and discussion

This paper provides a qualitative and then quantitative approach for dividing the cycle of an infectious disease outbreak into five stages: introduction, early infectors, early majority, late majority, and laggards. We show how implementing countermeasures in different stages causes different evolutionary dynamics for new infected over the whole cycle. Results are consistent and clear: Implement countermeasures earlier can always achieve better performances. Moreover, when to implement countermeasures can be more important than the countermeasures themselves. Implementing countermeasures in the early stages enjoy two-fold benefits: less societal and economic cost and fewer total infected in the end.

This paper therefore argues that it is not only necessary but also mandatory to employ different focuses in each stage over the life cycle of an infectious disease outbreak. Our systematic approach can be viewed as analogous to how we treat cancer. In the early stages, we need to focus on prevention, early detection, and strict countermeasure (e.g., isolation and lockdown) for controlling an epidemic. It is better safe (i.e., stricter countermeasures) than sorry (i.e., let the virus spread out). Similarly, for cancer treatment in its early stages, we focus on prevention, early detection, and strict countermeasure (e.g., surgical removal and precise radiation therapy on a small tumor area) since a tumor is still small and has not spread out to other tissues. There are two reasons why we should implement responsive and strict countermeasures in the early stages. The countermeasures are very effective (e.g., achieving zero COVID in China in the early stages), and the earlier the more total infected reduction over the whole cycle. The economic and societal burden for implementing countermeasures is relatively small due to limited affected areas, and the earlier the less burden. Both reasons change to the opposite in the late stages. The strategic focuses in the late stages become more delicate and balanced for two reasons: the same countermeasures become much less effective, and the society bears a much heavier burden. Strict countermeasures may become unnecessary, and we need to think about how to live with the infectious disease. We need to take a more comprehensive view of cancer treatment in the late stages and consider its impact on the whole body because the tumor has grown bigger and spread to other tissues. Overall, it is always better responsive and safe than sorry in the early stages because benefits are exponentially large while costs and efforts are extremely limited. Countermeasures in the late stages will not make much difference regarding total infected, while only radically increase extra burdens on society and economy.

When to reopen the business works in the opposite direction: The cumulative infected will increase more if we reopen businesses in the early stages. In addition, when a vaccine is available, we need to move these vaccinated individuals from susceptible to recovered (i.e., immuned) or lower the transmission rate. The rest of analysis is the same. Moreover, the distribution of vaccines should be along the critical diffusion paths where individuals who most likely get infected should receive vaccinations first.

Regarding asymptomatic infections, we must realize that there are more asymptomatic and underreported cases in the early stages, characterized by the unfamiliar public with a new infectious disease, less testing capacity and practice, and more chaotic communications. We tend to miss the asymptomatic cases and thus underestimate the severity of the new infectious disease. We can apply the literature results in asymptomatic cases [19] to obtain more accurate estimates. The opposites of the above characteristics are true in the late stages. Our countermeasures become more reasonable and control policies become more rational in the late stages.

## Supporting information

S1 File (DOCX)

## References

[1] Tam KM, Walker N, Moreno J. Effect of mitigation measures on the spreading of COVID-19 in hard-hit states in the U.S. PLoS ONE. 2020; 15(11):e0240877. 10.1371/journal. pone.0240877. 33141823 PMC7608876

[2] Quaas MF, Meya JN, Schenk H, Bos B, Drupp MA, Requate T. The social cost of contacts: Theory and evidence for the first wave of the COVID-19 pandemic in Germany. PLoS ONE. 2021;16(3): e0248288. 10.1371/journal. pone.0248288. 33740007 PMC7978372

[3] Kim SR, Kung T, Abdelmalek M. Trust, testing and tracing: How South Korea succeeded where the US stumbled in coronavirus response: As US tops 60k deaths, South Korea marks first day without a new local case. 2020; Available at ABC news, https://abcnews.go.com/Health/trust-testing-tracing-south-korea-succeeded-us-stumbled/story?id=70433504 (accessed date May 10, 2020).

[4] Goel S, Anderson A, Hofman J, Watts DJ. The structural virality of online diffusion. Management Science. 2016; 62: 180–196.

[5] Angst CM, Agarwal R, Sambamurthy V, Kelley K. Social contagion and information technology diffusion: The adoption of electronic medical records in U.S. hospitals. Management Science. 2010; 56:1219–1241.

[6] Bass FM. A new product growth for model consumer durables. Management Science. 1969; 15(5):215–227.

[7] Bass FM. Comments on “A new product growth for model consumer durables: The bass model.” Management Science. 2004; 50(12):1833–1840.

[8] Walker JL. The diffusion of innovations among the American States. The American Political Science Review. 1969; 63:880–99.

[9] Rogers EM. Diffusion of innovation. 5th ed. New York, NY: The Free Press; 2003.

[10] Sherlaw W, Rayde J. Why the French did not choose to panic: A dynamic analysis of the public response to the influenza pandemic. Sociology of Health and Illness. 2013; 35:332–344. 23030815 10.1111/j.1467-9566.2012.01525.x

[11] Robertsonm TS. The process of innovation and the diffusion of innovation. Journal of Marketing. 1967; 31:14–19.

[12] Agyeman P, Desgrandchamps D, Vaudaux B, Berger C, Diana A, Heininger U, Siegrist C-A, Aebi C. Interpretation of primary care physicians’ attitude regarding rotavirus immunisation using diffusion of innovation theories. Vaccine. 2009; 27:4771–4775. 19540950 10.1016/j.vaccine.2009.05.097

[13] Holmdahl I, Buckee C. Wrong but useful—What Covid-19 epidemiologic models can and cannot tell us. New England Journal of Medicine. 2020; 383:303–305. 32412711 10.1056/NEJMp2016822

[14] Thompson RN. Epidemiological models are important tools for guiding COVID-19 interventions. BMC Medicine. 2020; 18:152, doi: 10.1186/s12916-020-01628-4 32448247 PMC7246085

[15] Siettos CI, Russo L. Mathematical modeling of infectious disease dynamics. Virulence. 2013; 4(4):295–306. 23552814 10.4161/viru.24041 PMC3710332

[16] Kermack WO, McKendrick AG. A contribution to the mathematical theory of epidemics. Proceedings of the Royal Society of London Series A. 1927; 115(772):700–721.

[17] Reppas AI, Spiliotis KG, Siettos CI. Epidemionics: From the host-host interactions to the systematic analysis of the emergent macroscopic dynamics of epidemic networks. Virulence. 2010; 1(4):338–349. 21178467 10.4161/viru.1.4.12196

[18] Siettos CI. Equation-Free multiscale computational analysis of individual-based epidemic dynamics on networks. Applied Mathematics and Computation. 2011; 218:324–336.

[19] Russo L, Anastassopoulou C, Tsakris A, Bifulco GN, Campana EF, Toraldo G, et al. Tracing day-zero and forecasting the COVID-19 outbreak in Lombardy, Italy: A compartmental modelling and numerical optimization approach. PLoS ONE. 2020; 15:e0240649. doi: 10.1371/journal.pone.0240649 33125393 PMC7598513

[20] Russel TW, Wu JT, Clifford S, Edmunds WJ, Kucharski AJ, Jit M. Effect of internationally imported cases on internal spread of COVID-19: A mathematical modelling study. Lancet Public Health. 2021; 6:e12–e20. 33301722 10.1016/S2468-2667(20)30263-2 PMC7801817

[21] Liu Z, Magal P, Webb G. Predicting the number of reported and unreported cases for the COVID-19 epidemics in China, South Korea, Italy, France, Germany and United Kingdom, Journal of Theoretical Biology. 2021; 509:110501. 32980371 10.1016/j.jtbi.2020.110501 PMC7516517

[22] Brown RA. A simple model for control of COVID-19 infections on an urban campus, Proceedings of the National Academy of Sciences. 2021; 118:e2105292118. 10.1073/pnas.2105292118. PMC8433581 34475214

[23] Bertozzi AL, Franco E, Mohler G, Short MB, Sledge D. The challenges of modeling and forecasting the spread of COVID-19. Proceedings of the National Academy of Sciences. 2020; 117(29):16732–16738. 10.1073/pnas.2006520117 PMC7382213 32616574

[24] Lin Q, Zhao S, Gao D, Lou Y, Yang S, Musa SS, et al. A conceptual model for the coronavirus disease 2019 (COVID-19) outbreak in Wuhan, China with individual reaction and governmental action. International Journal of Infectious Diseases. 2020; 93:211–216. 32145465 10.1016/j.ijid.2020.02.058 PMC7102659

