# Supplementary material for: Mitigating the outbreak of an infectious disease over its life cycle: A diffusion-based approach
Source: PLoS One. 2023 Jan 26;18(1):e0280429. doi: 10.1371/journal.pone.0280429 (PMC9879393; doi:10.1371/journal.pone.0280429)
Supplement: S1 File — (DOCX) [file pone.0280429.s001.docx]

S1 File

We download daily cases of COVID-19 in Germany from Our World in Data [1]. The dataset’s daily new cases in Germany start on January 27, 2020. But most daily new cases are zeros in January and February 2020. So, we use daily new cases from March 1 to May 31 (i.e., 92 periods) to show the first wave in Germany.

The reproduction number tends to vary substantially in the initial periods of COVID-19 because of unreliable reporting, the small data time step (i.e., daily), and different countermeasures. We here choose a novel, statistically robust method by Cori et al. [2] for estimating the instantaneous reproduction number, where $\text{R}_{t,\tau}$ is over a time window of length $\tau=7$ ending at time *t*, depending on the number of incident cases in the time window [*t − τ* + 1, *t*], because the reported data typically demonstrate a weekly pattern.

Figure S1 shows daily cases in Germany and our estimated instantaneous reproduction number using Cori et al.’s tool [2]. The estimated instantaneous reproduction number drops from about 3 to under 1, fluctuating around 0.75. Note that more than 99% of the German population were still susceptible to COVID-19 after this first wave [3].

**S1. Fig Daily new COVID-19 cases in Germany from March to May 2020 and our estimated instantaneous reproduction number.**

Thus, we use the same base model: $\gamma=0.125$ and $\text{R}_{0}$ $=3$ (or equivalently $\beta=0.375$) with the following initial values in period 0: ${S_{0}=0.999999, I}_{0}=0.000001,$ and $R_{0}=0$. Germany implemented countermeasures around the middle of March 2020 [3-4]. We lower the transmission rate to $\beta$= 0.375/4 = 0.09375 (i.e., $\text{R}_{0}$ $=0.75$), starting in periods 5, 10, 15, 20, and 25, respectively, to reflect the timing value.

If we implement countermeasures earlier, new infected are depressed earlier and are lowered in peak, shown in Figures S2a and S2b. The new infected change exponentially in these initial periods. If we implement countermeasures in period 5, the new infected peak is 7.32x10^-7^ in period 4, while the new infected peak is 6.35x10^-5^ in period 24 for countermeasures implemented in period 25. The difference is almost 100-fold. The cumulative new infected demonstrates the same relationship: the earlier countermeasures, the more total infected reduction.

**S2a. Fig New infected of lower transmission in different starting periods.**

**S2b. Fig Cumulative new infected of lower transmission in different starting periods.**

1. Our World in Data. Germany: Coronavirus pandemic country profile; 2022. Available from: https://ourworldindata.org/coronavirus/country/germany.
2. Cori A, Ferguson NM, Fraser C, Cauchemez S. A new framework and software to estimate time-varying reproduction numbers during epidemics. American Journal of Epidemiology. 2013; 178(9):1505–1512.
3. Quaas MF, Meya JN, Schenk H, Bos B, Drupp MA, Requate T. The social cost of contacts: Theory and evidence for the first wave of the COVID-19 pandemic in Germany. PLoS ONE. 2021; 16(3):e0248288. https://doi.org/10.1371/journal. pone.0248288.
4. Liu Z, Magal P, Webb G. Predicting the number of reported and unreported cases for the COVID-19 epidemics in China, South Korea, Italy, France, Germany and United Kingdom, Journal of Theoretical Biology. 2021; 509:110501.
