# Supplementary material for: Mitigating the outbreak of an infectious disease over its life cycle: A diffusion-based approach
Source: PLoS One. 2023 Jan 26;18(1):e0280429. doi: 10.1371/journal.pone.0280429 (PMC9879393; doi:10.1371/journal.pone.0280429)
Supplement: Attachment — Submitted filename: Response to Reviewers.docx [file pone.0280429.s002.docx]

**Mitigating the outbreak of an infectious disease over its life cycle: A diffusion-based Approach**

We appreciate your help. Please see our below responses to the Editor and Reviewer 1.

**Editor’s comments**

**Furthermore I have reviewed the manuscript by myself and I find that you work can be potentially accepted. My main concern if that in your model you do not take into account the compartment of asymptomatics who have been proven to equally important to the transmission process of the disease. I do not suggest that the authors should change their model but a thorough discussion about that is necessary. Among the works that have introduced this in the modelling of COVID19 dynamics, the authors can refer to the following sources:**

**Russo et al. (2020) Tracing day-zero and forecasting the COVID-19 outbreak in Lombardy, Italy: a compartmental modelling and numerical optimization approach. PLoS ONE 15, e0240649.**

**Russel et al. (2021) Effect of internationally imported cases on internal spread of COVID-19: a mathematical modelling study, Lancet Bublic Health 6, e12-e20**

**Liu, et al. (2021) Predicting the number of reported and unreported cases for the COVID-19 epidemics in China, South Korea, Italy, France, Germany and United Kingdom, Journal of Theoretical Biology, 509, 110501.**

**Brown (2021) A simple model for control of COVID-19 infections on an urban campus, PNAS 118, e2105292118.**

Following your above suggestions and also from Reviewer 1, we add the following:

Mathematical models are vital for fighting epidemics. [1-2, 13-15]. Siettos and Russo [15] provide a comprehensive review of three main approaches for the surveillance and modeling of infectious disease dynamics: (1) statistical methods for surveillance of outbreaks and identification of spatial patterns in real epidemics, (2) mathematical models within the context of dynamical systems used to forecast the evolution of a “hypothetical” or on-going epidemic spread, and (3) machine learning/expert methods for the forecasting of the evolution of an ongoing epidemic. Also, importantly, Siettos and Russo [15] present basic relevant concepts underpinning their implementation and practice with representative works. The celebrated Kermack and McKendrick [16] model (i.e., the susceptible-infected-recovered (SIR) model) is the basis of mathematical/mechanistic state-space models.

Mechanistic models simulate the ways how infectious diseases spread and can fit many future transmission scenarios by adjusting assumptions and model parameters. [13-14] Mechanistic models can make long-term predictions. Because epidemiologic data are evolving and we face a lot of uncertainties and dissimilar reactions, we need to carry out sensitivity analyses to figure out the converging patterns.

Controlling an epidemic appears to be one major challenge nowadays. Researchers have made improvements from various perspectives. Reppas et al. [17] apply a novel equation-free computational framework for multi-scale analysis to effectively analyze the macroscopic emergent behavior of complex epidemic models. They thus solve a critical issue that bridges the diverse space and time scales from the microscopic scale, including the immune mechanisms, host-microbe, and host-host interactions, to the macroscopic population scale with an emergent epidemic. Siettos [18] exploits the equation-free approach and optimization methods to bridge detailed individual-based epidemic models with coarse-grained, system-level analysis within a pair-wise representation perspective. Siettos [18] can therefore perform steady state computations, stability computations, and continuation and numerical bifurcation analysis of the complex-emergent dynamics while bypassing the need of analytical derivation of closures for the macroscopic-level equations.

Some researchers also investigate the important role of asymptomatic infections in an epidemic. Russo et al. [19] provide prompt estimates for the day-zero of the COVID-19 outbreak in Lombardy, Italy; the actual number of asymptomatic infected cases in the total population until March 8, 2020; and the basic and the effective reproduction numbers. Russo et al. [19] model two compartments of infected individuals: (1) asymptomatic or experiencing very mild symptoms and (2) mild to severe symptoms. Note that COVID-29 was in the early stages from January to March 2020 in Italy. “As mentioned in the methodology, within the first period of the outbreak the number of confirmed cases was approximately equal to the number of hospitalized cases, i.e. there was no sampling strategy. Furthermore, as also reported for the later period, tests were conducted only for those who seeked medical care and had symptoms like fever and coughing. Thus, people who did not seek for medical attention were tested very scarcely” [19]. Most asymptomatic cases were not reported in the early stages of COVID-19. Russo et al. [19] successfully bridge the gap between asymptomatic and symptomatic cases and thus provide more accurate estimates for the effective control policies in real time. Russell et al. [20] accommodate asymptomatic cases to adjust reported cases such that they can correctly investigate how internationally imported cases cause an internal spread of COVID-19, due to different international travel restrictions. Liu et al. [21] incorporate asymptomatic and symptomatic, reported and unreported data to model the COVID-19 transmission dynamics in China, South Korea, Italy, France, Germany, and the United Kingdom. Liu et al. [21] evaluate both the early exponential growth phase before the government implementation of major control measures and the next phase when these measures result in a time-dependent exponentially decreasing number of cases. Brown [22] presents a customized susceptible, exposed, infected, and recovered compartmental model for illustrating the asymptomatic spread of COVID-19 at Boston University. Brown [22] finds that the spread of COVID-19 is related to the exogenous infection rate in the community and is reduced by more frequent testing, effective contact tracing, and vaccination.

Regarding asymptomatic infections, we must realize that there are more asymptomatic and underreported cases in the early stages, characterized by the unfamiliar public with a new infectious disease, less testing capacity and practice, and more chaotic communications. We tend to miss the asymptomatic cases and thus underestimate the severity of the new infectious disease. We can apply the literature results in asymptomatic cases [19] to obtain more accurate estimates. The opposites of the above characteristics are true in the late stages. Our countermeasures become more reasonable and control policies become more effective in the late stages.

15. Siettos CI, Russo L. Mathematical modeling of infectious disease dynamics. Virulence. 2013; 4(4):295–306.

17. Reppas AI, Spiliotis KG, Siettos CI. Epidemionics: From the host-host interactions to the systematic analysis of the emergent macroscopic dynamics of epidemic networks. Virulence. 2010; 1(4):338-349.

18. Siettos CI. Equation-Free multiscale computational analysis of individual-based epidemic dynamics on networks. Applied Mathematics and Computation. 2011; 218:324–336.

19. Russo L, Anastassopoulou C, Tsakris A, Bifulco GN, Campana EF, Toraldo G, Siettos C. Tracing day-zero and forecasting the COVID-19 outbreak in Lombardy, Italy: A compartmental modelling and numerical optimization approach. PLoS ONE. 2020; 15:e0240649. https://doi.org/10.1371/journal.pone.0240649.

20. Russel TW, Wu JT, Clifford S, Edmunds WJ, Kucharski AJ, Jit M. Effect of internationally imported cases on internal spread of COVID-19: A mathematical modelling study. Lancet Public Health. 2021; 6:e12-e20.

21. Liu Z, Magal P, Webb G. Predicting the number of reported and unreported cases for the COVID-19 epidemics in China, South Korea, Italy, France, Germany and United Kingdom, Journal of Theoretical Biology. 2021; 509:110501.

22. Brown RA. A simple model for control of COVID-19 infections on an urban campus, Proceedings of the National Academy of Sciences. 2021; 118:e2105292118. https://doi.org/10.1073/pnas.2105292118.

**Reviewer 1:**

We have revised our manuscript by following your helpful suggestions. Please see below for our responses in a point-by-point format. Thank you for your valuable comments.

**“The authors propose a new computational approach to investigate a possible control of the COVID pandemic. Their new approach is based on the essential elements of diffusion theory with a combination of discrete SIR models. The discrete SIR model predicts proxy a normal distribution of daily new infections with respect to time. With this assumption, ones can divide the whole live cycle of COVID into 5-periods: Introduction (infection<2.5%), Early infectors (2.5%<infection<16%), Early majority (16%<infection<50%), etc. This separation corresponds to a specific time, e.g. 1.25% is the 41 time step (day) from the beginning of infections.**

**Having the periods that characterized the cycle of the COVID pandemic, the authors change the model's parameters in different periods aspiring to imitate countermeasures e.g. lockdown.**

**I find their approximation interesting but straightforward, suggesting that major review changes should be made in the review process.**

**The authors have to revise their paper by taking into account the following points when revising their manuscript:**

1. **abstract**

**Should give the main question of the research. The phrase: ‘‘… it is always better safe than sorry with a focus on time/speed rather than cost because same countermeasures depress infections most’’. it is not clear what the authors suggest.”**

We add the following:

We aim to answer the following three research questions: How to divide an infectious disease cycle into different stages? How do countermeasures in different stages cause different evolutionary dynamics? How to set up appropriate countermeasure strategies in different stages?

For appropriate strategic focuses in different stages, what we try to say is that these differentiated strategies are like how we treat cancer:

Our systematic approach can be viewed as analogous to how we treat cancer. In the early stages, we need to focus on prevention, early detection, and strict countermeasure (e.g., isolation and lockdown) for controlling an epidemic. It is better safe (i.e., stricter countermeasures) than sorry (i.e., let the virus spread out). (Similarly, for cancer treatment in its early stages, we focus on prevention, early detection, and strict countermeasure (e.g., surgical removal and precise radiation therapy on a small tumor area) since a tumor is still small and has not spread out to other tissues.) There are two reasons why we should implement responsive and strict countermeasures in the early stages. The countermeasures are very effective (e.g., achieving zero COVID in China in the early stages), and the earlier the more total infected reduction over the whole cycle. The economic and societal burden for implementing countermeasures is relatively small due to limited affected areas, and the earlier the less burden. Both reasons change to the opposite in the late stages. The strategic focuses in the late stages become more delicate and balanced for two reasons: the same countermeasures become much less effective, and the society bears a much heavier burden. Strict countermeasures may become unnecessary, and we need to think about how to live with the infectious disease. (We need to take a more comprehensive view of cancer treatment in the late stages and consider its impact on the whole body because the tumor has grown bigger and spread to other tissues.)

We revise our abstract and also add our above comments in the discussion section.

**“‘’…infected variable’’ there is no infected variable, please rephrase.”**

We delete the word “variable” and rephrase the sentence to:

In particular, we apply the specific values of the newly infected to quantitatively divide an outbreak cycle into stages.

**“2. Introduction**

**The part of Epidemic models is poor. The authors should refer to:**

**Siettos, Equation-Free multiscale computational analysis of individual-based epidemic dynamics on networks, App. Math. Comp.218. 324 (2011)**

**epidemionics**

**Andreas I. Reppas, Konstantinos G. Spiliotis & Constantinos I. Siettos (2010) Epidemionics: From the host-host interactions to the systematic analysis of the emergent macroscopic dynamics of epidemic networks, Virulence, 1:4, 338-349,**

**Constantinos I. Siettos & Lucia Russo (2013) Mathematical modeling of infectious disease dynamics, Virulence, 4:4, 295-306,**

**For completeness, the authors should increase this part.”**

Following your above suggestions and also from the editor, we add the following:

Mathematical models are vital for fighting epidemics. [1-2, 13-15]. Siettos and Russo [15] provide a comprehensive review of three main approaches for the surveillance and modeling of infectious disease dynamics: (1) statistical methods for surveillance of outbreaks and identification of spatial patterns in real epidemics, (2) mathematical models within the context of dynamical systems used to forecast the evolution of a “hypothetical” or on-going epidemic spread, and (3) machine learning/expert methods for the forecasting of the evolution of an ongoing epidemic. Also, importantly, Siettos and Russo [15] present basic relevant concepts underpinning their implementation and practice with representative works. The celebrated Kermack and McKendrick [16] model (i.e., the susceptible-infected-recovered (SIR) model) is the basis of mathematical/mechanistic state-space models.

Mechanistic models simulate the ways how infectious diseases spread and can fit many future transmission scenarios by adjusting assumptions and model parameters. [13-14] Mechanistic models can make long-term predictions. Because epidemiologic data are evolving and we face a lot of uncertainties and dissimilar reactions, we need to carry out sensitivity analyses to figure out the converging patterns.

Controlling an epidemic appears to be one major challenge nowadays. Researchers have made improvements from various perspectives. Reppas et al. [17] apply a novel equation-free computational framework for multi-scale analysis to effectively analyze the macroscopic emergent behavior of complex epidemic models. They thus solve a critical issue that bridges the diverse space and time scales from the microscopic scale, including the immune mechanisms, host-microbe, and host-host interactions, to the macroscopic population scale with an emergent epidemic. Siettos [18] exploits the equation-free approach and optimization methods to bridge detailed individual-based epidemic models with coarse-grained, system-level analysis within a pair-wise representation perspective. Siettos [18] can therefore perform steady state computations, stability computations, and continuation and numerical bifurcation analysis of the complex-emergent dynamics while bypassing the need of analytical derivation of closures for the macroscopic-level equations.

Some researchers also investigate the important role of asymptomatic infections in an epidemic. Russo et al. [19] provide prompt estimates for the day-zero of the COVID-19 outbreak in Lombardy, Italy; the actual number of asymptomatic infected cases in the total population until March 8, 2020; and the basic and the effective reproduction numbers. Russo et al. [19] model two compartments of infected individuals: (1) asymptomatic or experiencing very mild symptoms and (2) mild to severe symptoms. Note that COVID-29 was in the early stages from January to March 2020 in Italy. “As mentioned in the methodology, within the first period of the outbreak the number of confirmed cases was approximately equal to the number of hospitalized cases, i.e. there was no sampling strategy. Furthermore, as also reported for the later period, tests were conducted only for those who seeked medical care and had symptoms like fever and coughing. Thus, people who did not seek for medical attention were tested very scarcely” [19]. Most asymptomatic cases were not reported in the early stages of COVID-19. Russo et al. [19] successfully bridge the gap between asymptomatic and symptomatic cases and thus provide more accurate estimates for the effective control policies in real time. Russell et al. [20] accommodate asymptomatic cases to adjust reported cases such that they can correctly investigate how internationally imported cases cause an internal spread of COVID-19, due to different international travel restrictions. Liu et al. [21] incorporate asymptomatic and symptomatic, reported and unreported data to model the COVID-19 transmission dynamics in China, South Korea, Italy, France, Germany, and the United Kingdom. Liu et al. [21] evaluate both the early exponential growth phase before the government implementation of major control measures and the next phase when these measures result in a time-dependent exponentially decreasing number of cases. Brown [22] presents a customized susceptible, exposed, infected, and recovered compartmental model for illustrating the asymptomatic spread of COVID-19 at Boston University. Brown [22] finds that the spread of COVID-19 is related to the exogenous infection rate in the community and is reduced by more frequent testing, effective contact tracing, and vaccination.

Regarding asymptomatic infections, we must realize that there are more asymptomatic and underreported cases in the early stages, characterized by the unfamiliar public with a new infectious disease, less testing capacity and practice, and more chaotic communications. We tend to miss the asymptomatic cases and thus underestimate the severity of the new infectious disease. We can apply the literature results in asymptomatic cases [19] to obtain more accurate estimates. The opposites of the above characteristics are true in the late stages. Our countermeasures become more reasonable and control policies become more effective in the late stages.

15. Siettos CI, Russo L. Mathematical modeling of infectious disease dynamics. Virulence. 2013; 4(4):295–306.

17. Reppas AI, Spiliotis KG, Siettos CI. Epidemionics: From the host-host interactions to the systematic analysis of the emergent macroscopic dynamics of epidemic networks. Virulence. 2010; 1(4):338-349.

18. Siettos CI. Equation-Free multiscale computational analysis of individual-based epidemic dynamics on networks. Applied Mathematics and Computation. 2011; 218:324–336.

19. Russo L, Anastassopoulou C, Tsakris A, Bifulco GN, Campana EF, Toraldo G, Siettos C. Tracing day-zero and forecasting the COVID-19 outbreak in Lombardy, Italy: A compartmental modelling and numerical optimization approach. PLoS ONE. 2020; 15:e0240649. https://doi.org/10.1371/journal.pone.0240649.

20. Russel TW, Wu JT, Clifford S, Edmunds WJ, Kucharski AJ, Jit M. Effect of internationally imported cases on internal spread of COVID-19: A mathematical modelling study. Lancet Public Health. 2021; 6:e12-e20.

21. Liu Z, Magal P, Webb G. Predicting the number of reported and unreported cases for the COVID-19 epidemics in China, South Korea, Italy, France, Germany and United Kingdom, Journal of Theoretical Biology. 2021; 509:110501.

22. Brown RA. A simple model for control of COVID-19 infections on an urban campus, Proceedings of the National Academy of Sciences. 2021; 118:e2105292118. https://doi.org/10.1073/pnas.2105292118.

**“ 3. Diffusion Theory**

**The diffusion theory is not something unique in the sense that we have different approaches in statistical mechanics, partial differential equations, physics etc. The standard normal type of distribution that results is a very elementary part of the diffusion process. So please give a more details description.”**

We add more details why we use the diffusion theory:

Terms in different categories such as “innovators” and “early adopters” are commonly used and well understood by the general public.

We have two key takeaways from the above categorization method. First, we must adopt a holistic view over the life cycle of diffusion. When we implement countermeasures, we need to consider their impact on that particular time/stage as well as on the whole life cycle to the very end of an epidemic. Second, different categories/stages tend to demonstrate their own characteristics. The diffusion theory literature has summarized three major traits: socioeconomic status, personality traits, and communication behavior. The diffusion of an infectious disease shows many similarities. When we implement countermeasures, we need to adopt distinct strategic focuses for different stages.

**“4. An SIR model for quantifying dynamics**

**The authors produce mathematical formulas without using them in the manuscript (210-215 lines in manuscript). Then what are the benefits?”**

We delete these formulas.

**“5. Quantifying dynamics in different stages and Sensitivity Analysis**

**The parameters: \beta ( lower transmission rate) and \gamma (quicker recovery rate) change in a discrete way in order to perform sensitivity analysis. For a constant \gamma, the authors should change the \beta parameter slowly (0.1<\beta<0.75). Then, the authors should present the maximum and the minimum value of the new infection variable (the last value on the cumulative diagram). In this way, the authors should produce upper and lower bounds for the cumulative number of the new infected variable with respect to the \beta parameter.”**

Thank you for this nice suggestion. We check our data again and find our sensitivity results are essentially same for different values of \gamma (recovery rate). So, we run our sensitivity with small changes of and add the following in our section of sensitivity analysis:

Among the three rows, i.e., same reproduction numbers but different recovery rates, the figure shapes are essentially the same. So, the recovery rate is trivial for the same $\text{R}_{0}$. The countermeasures benefits are larger if the starting implementation period of such a change is earlier. Promptness is very important.

We then further run small changes of the transmission rates to reflect different levels of countermeasures, e.g., lockdown, business and school closing, partial opening, etc. We use our base model with $\gamma=0.125$ and $\text{R}_{0}$ $=3$ (or $\beta=0.375$) as the starting point. We lower the transmission rate to 0.35, 0.3, 0.25, 0.2, 0.15, 0.1, and 0.05, respectively. We have the same finding as before: we will have a more total infected reduction if we implement countermeasures earlier. In addition, stricter countermeasures will have a larger impact on the total infected reduction. The relationship is monotonic. If we implement countermeasures too late, the total infected over the whole cycle is almost the same as in our base model, with not much total infected reduction. There is the upper bound for the cumulative number of the new infected variable, 94.83% in our base model. Our lowest total infected is 1.60%, caused by the lowest $\beta=0.05$ in the introduction stage. 1.60% is the lower bound for the cumulative number of the new infected variable in these scenarios.

**Fig 5. The cumulative infected (i.e., the recovered fractions or total infected) with different countermeasures on transmission rates (**$\beta$ **beta).**

|  | β 0.05 | β 0.1 | β 0.15 | β 0.2 | β 0.25 | β 0.3 | β 0.35 |
| --- | --- | --- | --- | --- | --- | --- | --- |
| Introduction | 0.0159991 | 0.0375706 | 0.338937 | 0.651204 | 0.805274 | 0.887074 | 0.933093 |
| Early infectors | 0.106659 | 0.191905 | 0.437115 | 0.676408 | 0.813192 | 0.889508 | 0.933544 |
| Early majority | 0.351739 | 0.461467 | 0.60961 | 0.74453 | 0.838251 | 0.897806 | 0.935153 |
| Late majority | 0.685754 | 0.740161 | 0.794855 | 0.844121 | 0.884579 | 0.915808 | 0.93908 |
| Laggards | 0.923031 | 0.926934 | 0.930871 | 0.934815 | 0.938739 | 0.942613 | 0.946411 |
| Base | 0.948273 | 0.948273 | 0.948273 | 0.948273 | 0.948273 | 0.948273 | 0.948273 |

We also add the following in our section of reopening businesses:

Among the three rows, i.e., same reproduction numbers but different recovery rates, the results are essentially the same. We now run more simulations with small transmission rate changes. We use our base model with $\gamma=0.125$ and $\text{R}_{0}$ $=3$ (or $\beta=0.375$) as the starting point. We increase the transmission rate to 0.4, 0.45, 0.5, 0.55, 0.65, 0.7, and 0.75, respectively.

We have the same finding as before, as shown in Fig 7: we will have a higher total infected if we reopen businesses earlier. In addition, a full opening (i.e., a higher transmission rate) will cause a higher total infected than a partial opening (i.e., a lower transmission rate). The relationship is monotonic. If we do not reopen businesses until very late, the total infected over the whole cycle is almost the same as in our base model, not much total infected increase. There is the lower bound for the cumulative number of the new infected variable, 94.83% in our base model. Our highest total infected is 99.91%, caused by the highest $\beta=0.75$ in the introduction stage. 99.91% is the upper bound for the cumulative number of the new infected variable in these scenarios.

**Fig 7. The cumulative infected (i.e., the recovered fractions or total infected) with different levels of reopening businesses.**

|  | β 0.4 | β 0.45 | β 0.5 | β 0.55 | β 0.6 | β 0.65 | β 0.7 | β 0.75 |
| --- | --- | --- | --- | --- | --- | --- | --- | --- |
| Introduction | 0.95995 | 0.975973 | 0.98564 | 0.991492 | 0.995023 | 0.997137 | 0.998387 | 0.999113 |
| Early infectors | 0.959688 | 0.975503 | 0.985167 | 0.99109 | 0.994712 | 0.996909 | 0.998228 | 0.999006 |
| Early majority | 0.958719 | 0.973716 | 0.983315 | 0.989472 | 0.993416 | 0.99593 | 0.997519 | 0.998512 |
| Late majority | 0.956116 | 0.968484 | 0.97743 | 0.983888 | 0.988543 | 0.991892 | 0.994294 | 0.996011 |
| Laggards | 0.950106 | 0.953677 | 0.957103 | 0.96037 | 0.963466 | 0.966386 | 0.969124 | 0.971683 |
| Base | 0.948273 | 0.948273 | 0.948273 | 0.948273 | 0.948273 | 0.948273 | 0.948273 | 0.948273 |

**“6. Application on real COVID data**

**As a suggestion to authors: I am wondering if the proposed methodology can be implemented retrospectively using real data of covid pandemic.**

**For example, the data (during the first wave in USA or Germany or worldwide) suggest a normal proxy distribution of the daily new infected cases (similar to the model). Under the assumption of strict lockdown, in the early stages, the authors should change the parameter \beta in order to fit and predict (retrospectively) the covid Pandemic for a small-time period. (If it works, it will strengthen the paper).”**

We add the first wave in Germany to demonstrate your suggestion in a supporting file:

S1 File

We download daily cases of COVID-19 in Germany from Our World in Data [1]. The dataset’s daily new cases in Germany start on January 27, 2020. But most daily new cases are zeros in January and February 2020. So, we use daily new cases from March 1 to May 31 (i.e., 92 periods) to show the first wave in Germany.

The reproduction number tends to vary substantially in the initial periods of COVID-19 because of unreliable reporting, the small data time step (i.e., daily), and different countermeasures. We here choose a novel, statistically robust method by Cori et al. [2] for estimating the instantaneous reproduction number, where $\text{R}_{t,\tau}$ is over a time window of length $\tau=7$ ending at time *t*, depending on the number of incident cases in the time window [*t − τ* + 1, *t*], because the reported data typically demonstrate a weekly pattern.

Figure S1 shows daily cases in Germany and our estimated instantaneous reproduction number using Cori et al.’s tool. The estimated instantaneous reproduction number drops from about 3 to under 1, fluctuating around 0.75. Note that more than 99% of the German population were still susceptible to COVID-19 after this first wave [3].

**S1. Fig Daily new COVID-19 cases in Germany from March to May 2020 and our estimated instantaneous reproduction number.**

|  | New cases | Instantaneous reproduction number |
| --- | --- | --- |
| 2020-03-01 | 51 |  |
| 2020-03-02 | 33 |  |
| 2020-03-03 | 38 |  |
| 2020-03-04 | 52 |  |
| 2020-03-05 | 109 |  |
| 2020-03-06 | 185 |  |
| 2020-03-07 | 150 | 3.34784 |
| 2020-03-08 | 163 | 2.64898 |
| 2020-03-09 | 265 | 2.59175 |
| 2020-03-10 | 184 | 2.2646 |
| 2020-03-11 | 271 | 2.14416 |
| 2020-03-12 | 802 | 2.65039 |
| 2020-03-13 | 693 | 2.51395 |
| 2020-03-14 | 733 | 2.34833 |
| 2020-03-15 | 1043 | 2.35826 |
| 2020-03-16 | 1174 | 2.27182 |
| 2020-03-17 | 1144 | 2.15404 |
| 2020-03-18 | 1042 | 1.97051 |
| 2020-03-19 | 2801 | 2.13541 |
| 2020-03-20 | 2958 | 2.20363 |
| 2020-03-21 | 2705 | 2.09008 |
| 2020-03-22 | 1948 | 1.82469 |
| 2020-03-23 | 4062 | 1.88033 |
| 2020-03-24 | 4764 | 1.95115 |
| 2020-03-25 | 4118 | 1.88542 |
| 2020-03-26 | 4954 | 1.74095 |
| 2020-03-27 | 5780 | 1.67589 |
| 2020-03-28 | 6294 | 1.66137 |
| 2020-03-29 | 3965 | 1.559 |
| 2020-03-30 | 4751 | 1.42597 |
| 2020-03-31 | 4615 | 1.30556 |
| 2020-04-01 | 5453 | 1.27761 |
| 2020-04-02 | 6156 | 1.25444 |
| 2020-04-03 | 6174 | 1.20776 |
| 2020-04-04 | 6082 | 1.15328 |
| 2020-04-05 | 5936 | 1.17892 |
| 2020-04-06 | 3677 | 1.11018 |
| 2020-04-07 | 3834 | 1.06234 |
| 2020-04-08 | 4003 | 1.0098 |
| 2020-04-09 | 4974 | 0.975711 |
| 2020-04-10 | 5323 | 0.958391 |
| 2020-04-11 | 4133 | 0.912675 |
| 2020-04-12 | 2821 | 0.838811 |
| 2020-04-13 | 2537 | 0.833179 |
| 2020-04-14 | 2082 | 0.810488 |
| 2020-04-15 | 2486 | 0.794179 |
| 2020-04-16 | 2866 | 0.757895 |
| 2020-04-17 | 3380 | 0.727465 |
| 2020-04-18 | 3609 | 0.75069 |
| 2020-04-19 | 2458 | 0.777722 |
| 2020-04-20 | 1775 | 0.782418 |
| 2020-04-21 | 1785 | 0.80427 |
| 2020-04-22 | 2237 | 0.825597 |
| 2020-04-23 | 2352 | 0.830478 |
| 2020-04-24 | 2337 | 0.807527 |
| 2020-04-25 | 2055 | 0.75978 |
| 2020-04-26 | 1737 | 0.75895 |
| 2020-04-27 | 1018 | 0.756293 |
| 2020-04-28 | 1144 | 0.757506 |
| 2020-04-29 | 1304 | 0.738524 |
| 2020-04-30 | 1478 | 0.721723 |
| 2020-05-01 | 1639 | 0.716055 |
| 2020-05-02 | 945 | 0.678156 |
| 2020-05-03 | 793 | 0.650345 |
| 2020-05-04 | 679 | 0.671098 |
| 2020-05-05 | 685 | 0.678159 |
| 2020-05-06 | 947 | 0.689859 |
| 2020-05-07 | 1284 | 0.714997 |
| 2020-05-08 | 1209 | 0.710826 |
| 2020-05-09 | 1251 | 0.787866 |
| 2020-05-10 | 667 | 0.808161 |
| 2020-05-11 | 357 | 0.79715 |
| 2020-05-12 | 933 | 0.860392 |
| 2020-05-13 | 798 | 0.865471 |
| 2020-05-14 | 933 | 0.838492 |
| 2020-05-15 | 913 | 0.822678 |
| 2020-05-16 | 620 | 0.760449 |
| 2020-05-17 | 583 | 0.784591 |
| 2020-05-18 | 342 | 0.819737 |
| 2020-05-19 | 513 | 0.780911 |
| 2020-05-20 | 797 | 0.814398 |
| 2020-05-21 | 745 | 0.813253 |
| 2020-05-22 | 460 | 0.758876 |
| 2020-05-23 | 638 | 0.799247 |
| 2020-05-24 | 431 | 0.80372 |
| 2020-05-25 | 289 | 0.824175 |
| 2020-05-26 | 432 | 0.836166 |
| 2020-05-27 | 362 | 0.764524 |
| 2020-05-28 | 353 | 0.706488 |
| 2020-05-29 | 741 | 0.822117 |
| 2020-05-30 | 738 | 0.883563 |
| 2020-05-31 | 286 | 0.863473 |

Thus, we use the same base model: $\gamma=0.125$ and $\text{R}_{0}$ $=3$ (or equivalently $\beta=0.375$) with the following initial values in period 0: ${S_{0}=0.999999, I}_{0}=0.000001,$ and $R_{0}=0$. Germany implemented countermeasures around the middle of March 2020 [3-4]. We lower the transmission rate to $\beta$= 0.375/4 = 0.09375 (i.e., $\text{R}_{0}$ $=0.75$), starting in periods 5, 10, 15, 20, and 25, respectively, to reflect the timing value.

If we implement countermeasures earlier, new infected are depressed earlier and are lowered in peak, shown in Figures S2a and S2b. The new infected change exponentially in these initial periods. If we implement countermeasures in period 5, the new infected peak is 7.32x10^-7^ in period 4, while the new infected peak is 6.35x10^-5^ in period 24 for countermeasures implemented in period 25. The difference is almost 100-fold. The cumulative new infected demonstrates the same relationship: the earlier countermeasures, the more total infected reduction.

**S2a. Fig New infected of lower transmission in different starting periods.**

|  | New infected 5 | New infected 10 | New infected 15 | New infected 20 | New infected 25 |
| --- | --- | --- | --- | --- | --- |
| 0 | 1e-06 | 1e-06 | 1e-06 | 1e-06 | 1e-06 |
| 1 | 3.75e-07 | 3.75e-07 | 3.75e-07 | 3.75e-07 | 3.75e-07 |
| 2 | 4.68749e-07 | 4.68749e-07 | 4.68749e-07 | 4.68749e-07 | 4.68749e-07 |
| 3 | 5.85936e-07 | 5.85936e-07 | 5.85936e-07 | 5.85936e-07 | 5.85936e-07 |
| 4 | 7.32419e-07 | 7.32419e-07 | 7.32419e-07 | 7.32419e-07 | 7.32419e-07 |
| 5 | 2.28881e-07 | 9.15523e-07 | 9.15523e-07 | 9.15523e-07 | 9.15523e-07 |
| 6 | 2.21728e-07 | 1.1444e-06 | 1.1444e-06 | 1.1444e-06 | 1.1444e-06 |
| 7 | 2.14799e-07 | 1.4305e-06 | 1.4305e-06 | 1.4305e-06 | 1.4305e-06 |
| 8 | 2.08086e-07 | 1.78812e-06 | 1.78812e-06 | 1.78812e-06 | 1.78812e-06 |
| 9 | 2.01584e-07 | 2.23514e-06 | 2.23514e-06 | 2.23514e-06 | 2.23514e-06 |
| 10 | 1.95284e-07 | 6.98477e-07 | 2.79391e-06 | 2.79391e-06 | 2.79391e-06 |
| 11 | 1.89181e-07 | 6.76649e-07 | 3.49237e-06 | 3.49237e-06 | 3.49237e-06 |
| 12 | 1.83269e-07 | 6.55502e-07 | 4.36542e-06 | 4.36542e-06 | 4.36542e-06 |
| 13 | 1.77542e-07 | 6.35017e-07 | 5.45673e-06 | 5.45673e-06 | 5.45673e-06 |
| 14 | 1.71994e-07 | 6.15171e-07 | 6.82083e-06 | 6.82083e-06 | 6.82083e-06 |
| 15 | 1.66619e-07 | 5.95946e-07 | 2.13148e-06 | 8.52591e-06 | 8.52591e-06 |
| 16 | 1.61412e-07 | 5.77322e-07 | 2.06486e-06 | 1.06572e-05 | 1.06572e-05 |
| 17 | 1.56368e-07 | 5.59279e-07 | 2.00032e-06 | 1.33212e-05 | 1.33212e-05 |
| 18 | 1.51481e-07 | 5.41801e-07 | 1.9378e-06 | 1.6651e-05 | 1.6651e-05 |
| 19 | 1.46747e-07 | 5.24868e-07 | 1.87723e-06 | 2.0813e-05 | 2.0813e-05 |
| 20 | 1.42161e-07 | 5.08465e-07 | 1.81856e-06 | 6.50376e-06 | 2.6015e-05 |
| 21 | 1.37718e-07 | 4.92574e-07 | 1.76172e-06 | 6.30041e-06 | 3.25169e-05 |
| 22 | 1.33415e-07 | 4.7718e-07 | 1.70665e-06 | 6.10342e-06 | 4.06433e-05 |
| 23 | 1.29245e-07 | 4.62268e-07 | 1.65331e-06 | 5.91258e-06 | 5.07995e-05 |
| 24 | 1.25206e-07 | 4.47821e-07 | 1.60163e-06 | 5.72771e-06 | 6.34923e-05 |
| 25 | 1.21294e-07 | 4.33825e-07 | 1.55157e-06 | 5.54862e-06 | 1.98386e-05 |
| 26 | 1.17503e-07 | 4.20267e-07 | 1.50308e-06 | 5.37513e-06 | 1.92177e-05 |
| 27 | 1.13831e-07 | 4.07133e-07 | 1.45609e-06 | 5.20706e-06 | 1.86161e-05 |
| 28 | 1.10274e-07 | 3.94409e-07 | 1.41058e-06 | 5.04424e-06 | 1.80334e-05 |
| 29 | 1.06828e-07 | 3.82083e-07 | 1.36649e-06 | 4.88651e-06 | 1.74689e-05 |
| 30 | 1.03489e-07 | 3.70142e-07 | 1.32378e-06 | 4.73371e-06 | 1.69221e-05 |
| 31 | 1.00255e-07 | 3.58574e-07 | 1.2824e-06 | 4.58569e-06 | 1.63923e-05 |
| 32 | 9.71219e-08 | 3.47368e-07 | 1.24232e-06 | 4.4423e-06 | 1.58792e-05 |
| 33 | 9.40868e-08 | 3.36512e-07 | 1.20349e-06 | 4.30339e-06 | 1.5382e-05 |
| 34 | 9.11465e-08 | 3.25995e-07 | 1.16587e-06 | 4.16882e-06 | 1.49005e-05 |
| 35 | 8.82981e-08 | 3.15807e-07 | 1.12943e-06 | 4.03846e-06 | 1.44339e-05 |
| 36 | 8.55387e-08 | 3.05937e-07 | 1.09413e-06 | 3.91217e-06 | 1.3982e-05 |
| 37 | 8.28656e-08 | 2.96376e-07 | 1.05993e-06 | 3.78983e-06 | 1.35442e-05 |
| 38 | 8.0276e-08 | 2.87113e-07 | 1.0268e-06 | 3.67132e-06 | 1.31201e-05 |
| 39 | 7.77673e-08 | 2.7814e-07 | 9.94701e-07 | 3.55651e-06 | 1.27093e-05 |
| 40 | 7.5337e-08 | 2.69448e-07 | 9.63609e-07 | 3.44529e-06 | 1.23113e-05 |
| 41 | 7.29826e-08 | 2.61027e-07 | 9.33489e-07 | 3.33755e-06 | 1.19258e-05 |
| 42 | 7.07019e-08 | 2.52869e-07 | 9.0431e-07 | 3.23317e-06 | 1.15524e-05 |
| 43 | 6.84924e-08 | 2.44966e-07 | 8.76043e-07 | 3.13206e-06 | 1.11906e-05 |
| 44 | 6.63519e-08 | 2.3731e-07 | 8.4866e-07 | 3.03412e-06 | 1.08401e-05 |
| 45 | 6.42784e-08 | 2.29894e-07 | 8.22133e-07 | 2.93923e-06 | 1.05007e-05 |
| 46 | 6.22696e-08 | 2.22709e-07 | 7.96435e-07 | 2.84731e-06 | 1.01718e-05 |
| 47 | 6.03236e-08 | 2.15749e-07 | 7.7154e-07 | 2.75827e-06 | 9.85324e-06 |
| 48 | 5.84385e-08 | 2.09006e-07 | 7.47423e-07 | 2.672e-06 | 9.54464e-06 |
| 49 | 5.66122e-08 | 2.02474e-07 | 7.2406e-07 | 2.58844e-06 | 9.24571e-06 |
| 50 | 5.4843e-08 | 1.96146e-07 | 7.01427e-07 | 2.50749e-06 | 8.95613e-06 |
| 51 | 5.31291e-08 | 1.90016e-07 | 6.79502e-07 | 2.42907e-06 | 8.67561e-06 |
| 52 | 5.14688e-08 | 1.84078e-07 | 6.58262e-07 | 2.3531e-06 | 8.40388e-06 |
| 53 | 4.98604e-08 | 1.78325e-07 | 6.37686e-07 | 2.27951e-06 | 8.14065e-06 |
| 54 | 4.83022e-08 | 1.72752e-07 | 6.17753e-07 | 2.20822e-06 | 7.88566e-06 |
| 55 | 4.67927e-08 | 1.67353e-07 | 5.98443e-07 | 2.13916e-06 | 7.63866e-06 |
| 56 | 4.53304e-08 | 1.62122e-07 | 5.79737e-07 | 2.07226e-06 | 7.39939e-06 |
| 57 | 4.39138e-08 | 1.57056e-07 | 5.61615e-07 | 2.00745e-06 | 7.16761e-06 |
| 58 | 4.25414e-08 | 1.52147e-07 | 5.4406e-07 | 1.94466e-06 | 6.94309e-06 |
| 59 | 4.1212e-08 | 1.47392e-07 | 5.27054e-07 | 1.88384e-06 | 6.72559e-06 |
| 60 | 3.99241e-08 | 1.42786e-07 | 5.10579e-07 | 1.82493e-06 | 6.51491e-06 |
| 61 | 3.86764e-08 | 1.38323e-07 | 4.94619e-07 | 1.76785e-06 | 6.31083e-06 |
| 62 | 3.74677e-08 | 1.34e-07 | 4.79158e-07 | 1.71256e-06 | 6.11313e-06 |
| 63 | 3.62968e-08 | 1.29812e-07 | 4.6418e-07 | 1.659e-06 | 5.92163e-06 |
| 64 | 3.51625e-08 | 1.25755e-07 | 4.4967e-07 | 1.60711e-06 | 5.73612e-06 |
| 65 | 3.40637e-08 | 1.21825e-07 | 4.35614e-07 | 1.55685e-06 | 5.55642e-06 |
| 66 | 3.29991e-08 | 1.18018e-07 | 4.21997e-07 | 1.50816e-06 | 5.38235e-06 |
| 67 | 3.19679e-08 | 1.14329e-07 | 4.08806e-07 | 1.46099e-06 | 5.21373e-06 |
| 68 | 3.09689e-08 | 1.10756e-07 | 3.96028e-07 | 1.41529e-06 | 5.05039e-06 |
| 69 | 3.00011e-08 | 1.07295e-07 | 3.83648e-07 | 1.37103e-06 | 4.89217e-06 |
| 70 | 2.90635e-08 | 1.03942e-07 | 3.71656e-07 | 1.32815e-06 | 4.7389e-06 |
| 71 | 2.81552e-08 | 1.00693e-07 | 3.60038e-07 | 1.28661e-06 | 4.59043e-06 |
| 72 | 2.72754e-08 | 9.75461e-08 | 3.48784e-07 | 1.24637e-06 | 4.44662e-06 |
| 73 | 2.6423e-08 | 9.44975e-08 | 3.37881e-07 | 1.20738e-06 | 4.3073e-06 |
| 74 | 2.55972e-08 | 9.15442e-08 | 3.2732e-07 | 1.16962e-06 | 4.17235e-06 |
| 75 | 2.47973e-08 | 8.86832e-08 | 3.17088e-07 | 1.13304e-06 | 4.04163e-06 |
| 76 | 2.40224e-08 | 8.59116e-08 | 3.07176e-07 | 1.0976e-06 | 3.915e-06 |
| 77 | 2.32716e-08 | 8.32266e-08 | 2.97574e-07 | 1.06327e-06 | 3.79234e-06 |
| 78 | 2.25444e-08 | 8.06255e-08 | 2.88272e-07 | 1.03002e-06 | 3.67352e-06 |
| 79 | 2.18398e-08 | 7.81057e-08 | 2.79261e-07 | 9.97799e-07 | 3.55842e-06 |
| 80 | 2.11573e-08 | 7.56647e-08 | 2.70532e-07 | 9.66591e-07 | 3.44693e-06 |
| 81 | 2.04961e-08 | 7.33e-08 | 2.62075e-07 | 9.36358e-07 | 3.33893e-06 |
| 82 | 1.98556e-08 | 7.10091e-08 | 2.53883e-07 | 9.07072e-07 | 3.23432e-06 |
| 83 | 1.92351e-08 | 6.87899e-08 | 2.45947e-07 | 8.78701e-07 | 3.13298e-06 |
| 84 | 1.8634e-08 | 6.664e-08 | 2.38259e-07 | 8.51217e-07 | 3.03481e-06 |
| 85 | 1.80517e-08 | 6.45573e-08 | 2.30811e-07 | 8.24593e-07 | 2.93972e-06 |
| 86 | 1.74875e-08 | 6.25397e-08 | 2.23596e-07 | 7.98802e-07 | 2.84761e-06 |
| 87 | 1.6941e-08 | 6.05851e-08 | 2.16607e-07 | 7.73818e-07 | 2.75839e-06 |
| 88 | 1.64116e-08 | 5.86917e-08 | 2.09836e-07 | 7.49614e-07 | 2.67196e-06 |
| 89 | 1.58987e-08 | 5.68574e-08 | 2.03277e-07 | 7.26168e-07 | 2.58823e-06 |
| 90 | 1.54019e-08 | 5.50804e-08 | 1.96922e-07 | 7.03455e-07 | 2.50713e-06 |
| 91 | 1.49206e-08 | 5.3359e-08 | 1.90767e-07 | 6.81453e-07 | 2.42858e-06 |
| 92 | 1.44543e-08 | 5.16914e-08 | 1.84803e-07 | 6.60138e-07 | 2.35248e-06 |

**S2b. Fig Cumulative new infected of lower transmission in different starting periods.**

|  | Cumulative new infected 5 | Cumulative new infected 10 | Cumulative new infected 15 | Cumulative new infected 20 | Cumulative new infected 25 |
| --- | --- | --- | --- | --- | --- |
| 0 | 1e-06 | 1e-06 | 1e-06 | 1e-06 | 1e-06 |
| 1 | 1.375e-06 | 1.375e-06 | 1.375e-06 | 1.375e-06 | 1.375e-06 |
| 2 | 1.84375e-06 | 1.84375e-06 | 1.84375e-06 | 1.84375e-06 | 1.84375e-06 |
| 3 | 2.42968e-06 | 2.42968e-06 | 2.42968e-06 | 2.42968e-06 | 2.42968e-06 |
| 4 | 3.1621e-06 | 3.1621e-06 | 3.1621e-06 | 3.1621e-06 | 3.1621e-06 |
| 5 | 3.39098e-06 | 4.07763e-06 | 4.07763e-06 | 4.07763e-06 | 4.07763e-06 |
| 6 | 3.61271e-06 | 5.22203e-06 | 5.22203e-06 | 5.22203e-06 | 5.22203e-06 |
| 7 | 3.82751e-06 | 6.65253e-06 | 6.65253e-06 | 6.65253e-06 | 6.65253e-06 |
| 8 | 4.0356e-06 | 8.44064e-06 | 8.44064e-06 | 8.44064e-06 | 8.44064e-06 |
| 9 | 4.23718e-06 | 1.06758e-05 | 1.06758e-05 | 1.06758e-05 | 1.06758e-05 |
| 10 | 4.43247e-06 | 1.13743e-05 | 1.34697e-05 | 1.34697e-05 | 1.34697e-05 |
| 11 | 4.62165e-06 | 1.20509e-05 | 1.69621e-05 | 1.69621e-05 | 1.69621e-05 |
| 12 | 4.80492e-06 | 1.27064e-05 | 2.13275e-05 | 2.13275e-05 | 2.13275e-05 |
| 13 | 4.98246e-06 | 1.33414e-05 | 2.67842e-05 | 2.67842e-05 | 2.67842e-05 |
| 14 | 5.15445e-06 | 1.39566e-05 | 3.3605e-05 | 3.3605e-05 | 3.3605e-05 |
| 15 | 5.32107e-06 | 1.45525e-05 | 3.57365e-05 | 4.21309e-05 | 4.21309e-05 |
| 16 | 5.48248e-06 | 1.51299e-05 | 3.78014e-05 | 5.27881e-05 | 5.27881e-05 |
| 17 | 5.63885e-06 | 1.56891e-05 | 3.98017e-05 | 6.61093e-05 | 6.61093e-05 |
| 18 | 5.79033e-06 | 1.62309e-05 | 4.17395e-05 | 8.27603e-05 | 8.27603e-05 |
| 19 | 5.93708e-06 | 1.67558e-05 | 4.36167e-05 | 0.000103573 | 0.000103573 |
| 20 | 6.07924e-06 | 1.72643e-05 | 4.54353e-05 | 0.000110077 | 0.000129588 |
| 21 | 6.21696e-06 | 1.77569e-05 | 4.7197e-05 | 0.000116377 | 0.000162105 |
| 22 | 6.35037e-06 | 1.8234e-05 | 4.89037e-05 | 0.000122481 | 0.000202748 |
| 23 | 6.47962e-06 | 1.86963e-05 | 5.0557e-05 | 0.000128393 | 0.000253548 |
| 24 | 6.60482e-06 | 1.91441e-05 | 5.21586e-05 | 0.000134121 | 0.00031704 |
| 25 | 6.72612e-06 | 1.95779e-05 | 5.37102e-05 | 0.00013967 | 0.000336879 |
| 26 | 6.84362e-06 | 1.99982e-05 | 5.52132e-05 | 0.000145045 | 0.000356097 |
| 27 | 6.95745e-06 | 2.04053e-05 | 5.66693e-05 | 0.000150252 | 0.000374713 |
| 28 | 7.06772e-06 | 2.07998e-05 | 5.80799e-05 | 0.000155296 | 0.000392746 |
| 29 | 7.17455e-06 | 2.11818e-05 | 5.94464e-05 | 0.000160183 | 0.000410215 |
| 30 | 7.27804e-06 | 2.1552e-05 | 6.07702e-05 | 0.000164916 | 0.000427137 |
| 31 | 7.3783e-06 | 2.19106e-05 | 6.20526e-05 | 0.000169502 | 0.00044353 |
| 32 | 7.47542e-06 | 2.22579e-05 | 6.32949e-05 | 0.000173944 | 0.000459409 |
| 33 | 7.5695e-06 | 2.25944e-05 | 6.44984e-05 | 0.000178248 | 0.000474791 |
| 34 | 7.66065e-06 | 2.29204e-05 | 6.56643e-05 | 0.000182417 | 0.000489691 |
| 35 | 7.74895e-06 | 2.32362e-05 | 6.67937e-05 | 0.000186455 | 0.000504125 |
| 36 | 7.83449e-06 | 2.35422e-05 | 6.78878e-05 | 0.000190367 | 0.000518107 |
| 37 | 7.91735e-06 | 2.38386e-05 | 6.89478e-05 | 0.000194157 | 0.000531651 |
| 38 | 7.99763e-06 | 2.41257e-05 | 6.99745e-05 | 0.000197828 | 0.000544772 |
| 39 | 8.0754e-06 | 2.44038e-05 | 7.09693e-05 | 0.000201385 | 0.000557481 |
| 40 | 8.15073e-06 | 2.46733e-05 | 7.19329e-05 | 0.00020483 | 0.000569792 |
| 41 | 8.22372e-06 | 2.49343e-05 | 7.28663e-05 | 0.000208168 | 0.000581718 |
| 42 | 8.29442e-06 | 2.51871e-05 | 7.37707e-05 | 0.000211401 | 0.00059327 |
| 43 | 8.36291e-06 | 2.54321e-05 | 7.46467e-05 | 0.000214533 | 0.000604461 |
| 44 | 8.42926e-06 | 2.56694e-05 | 7.54954e-05 | 0.000217567 | 0.000615301 |
| 45 | 8.49354e-06 | 2.58993e-05 | 7.63175e-05 | 0.000220506 | 0.000625802 |
| 46 | 8.55581e-06 | 2.6122e-05 | 7.71139e-05 | 0.000223354 | 0.000635974 |
| 47 | 8.61613e-06 | 2.63378e-05 | 7.78855e-05 | 0.000226112 | 0.000645827 |
| 48 | 8.67457e-06 | 2.65468e-05 | 7.86329e-05 | 0.000228784 | 0.000655371 |
| 49 | 8.73118e-06 | 2.67493e-05 | 7.9357e-05 | 0.000231372 | 0.000664617 |
| 50 | 8.78603e-06 | 2.69454e-05 | 8.00584e-05 | 0.00023388 | 0.000673573 |
| 51 | 8.83916e-06 | 2.71354e-05 | 8.07379e-05 | 0.000236309 | 0.000682249 |
| 52 | 8.89063e-06 | 2.73195e-05 | 8.13961e-05 | 0.000238662 | 0.000690653 |
| 53 | 8.94049e-06 | 2.74978e-05 | 8.20338e-05 | 0.000240942 | 0.000698793 |
| 54 | 8.98879e-06 | 2.76706e-05 | 8.26516e-05 | 0.00024315 | 0.000706679 |
| 55 | 9.03558e-06 | 2.78379e-05 | 8.325e-05 | 0.000245289 | 0.000714318 |
| 56 | 9.08091e-06 | 2.8e-05 | 8.38298e-05 | 0.000247361 | 0.000721717 |
| 57 | 9.12483e-06 | 2.81571e-05 | 8.43914e-05 | 0.000249369 | 0.000728885 |
| 58 | 9.16737e-06 | 2.83093e-05 | 8.49354e-05 | 0.000251313 | 0.000735828 |
| 59 | 9.20858e-06 | 2.84566e-05 | 8.54625e-05 | 0.000253197 | 0.000742553 |
| 60 | 9.2485e-06 | 2.85994e-05 | 8.59731e-05 | 0.000255022 | 0.000749068 |
| 61 | 9.28718e-06 | 2.87378e-05 | 8.64677e-05 | 0.00025679 | 0.000755379 |
| 62 | 9.32465e-06 | 2.88718e-05 | 8.69468e-05 | 0.000258502 | 0.000761492 |
| 63 | 9.36094e-06 | 2.90016e-05 | 8.7411e-05 | 0.000260161 | 0.000767414 |
| 64 | 9.39611e-06 | 2.91273e-05 | 8.78607e-05 | 0.000261769 | 0.00077315 |
| 65 | 9.43017e-06 | 2.92491e-05 | 8.82963e-05 | 0.000263325 | 0.000778707 |
| 66 | 9.46317e-06 | 2.93672e-05 | 8.87183e-05 | 0.000264834 | 0.000784089 |
| 67 | 9.49514e-06 | 2.94815e-05 | 8.91271e-05 | 0.000266295 | 0.000789303 |
| 68 | 9.52611e-06 | 2.95922e-05 | 8.95231e-05 | 0.00026771 | 0.000794353 |
| 69 | 9.55611e-06 | 2.96995e-05 | 8.99068e-05 | 0.000269081 | 0.000799245 |
| 70 | 9.58517e-06 | 2.98035e-05 | 9.02784e-05 | 0.000270409 | 0.000803984 |
| 71 | 9.61333e-06 | 2.99042e-05 | 9.06385e-05 | 0.000271696 | 0.000808574 |
| 72 | 9.6406e-06 | 3.00017e-05 | 9.09873e-05 | 0.000272942 | 0.000813021 |
| 73 | 9.66702e-06 | 3.00962e-05 | 9.13252e-05 | 0.000274149 | 0.000817328 |
| 74 | 9.69262e-06 | 3.01878e-05 | 9.16525e-05 | 0.000275319 | 0.000821501 |
| 75 | 9.71742e-06 | 3.02764e-05 | 9.19696e-05 | 0.000276452 | 0.000825542 |
| 76 | 9.74144e-06 | 3.03624e-05 | 9.22767e-05 | 0.00027755 | 0.000829457 |
| 77 | 9.76471e-06 | 3.04456e-05 | 9.25743e-05 | 0.000278613 | 0.00083325 |
| 78 | 9.78726e-06 | 3.05262e-05 | 9.28626e-05 | 0.000279643 | 0.000836923 |
| 79 | 9.8091e-06 | 3.06043e-05 | 9.31418e-05 | 0.000280641 | 0.000840482 |
| 80 | 9.83025e-06 | 3.068e-05 | 9.34124e-05 | 0.000281607 | 0.000843929 |
| 81 | 9.85075e-06 | 3.07533e-05 | 9.36744e-05 | 0.000282544 | 0.000847268 |
| 82 | 9.87061e-06 | 3.08243e-05 | 9.39283e-05 | 0.000283451 | 0.000850502 |
| 83 | 9.88984e-06 | 3.08931e-05 | 9.41743e-05 | 0.000284329 | 0.000853635 |
| 84 | 9.90847e-06 | 3.09597e-05 | 9.44125e-05 | 0.000285181 | 0.00085667 |
| 85 | 9.92653e-06 | 3.10243e-05 | 9.46433e-05 | 0.000286005 | 0.000859609 |
| 86 | 9.94401e-06 | 3.10868e-05 | 9.48669e-05 | 0.000286804 | 0.000862457 |
| 87 | 9.96095e-06 | 3.11474e-05 | 9.50836e-05 | 0.000287578 | 0.000865215 |
| 88 | 9.97737e-06 | 3.12061e-05 | 9.52934e-05 | 0.000288327 | 0.000867887 |
| 89 | 9.99327e-06 | 3.1263e-05 | 9.54967e-05 | 0.000289054 | 0.000870476 |
| 90 | 1.00087e-05 | 3.1318e-05 | 9.56936e-05 | 0.000289757 | 0.000872983 |
| 91 | 1.00236e-05 | 3.13714e-05 | 9.58844e-05 | 0.000290439 | 0.000875411 |
| 92 | 1.0038e-05 | 3.14231e-05 | 9.60692e-05 | 0.000291099 | 0.000877764 |

1. Our World in Data. Germany: Coronavirus pandemic country profile; 2022. Available from: https://ourworldindata.org/coronavirus/country/germany.
2. Cori A, Ferguson NM, Fraser C, Cauchemez S. A new framework and software to estimate time-varying reproduction numbers during epidemics. American Journal of Epidemiology. 2013; 178(9):1505–1512.
3. Quaas MF, Meya JN, Schenk H, Bos B, Drupp MA, Requate T. The social cost of contacts: Theory and evidence for the first wave of the COVID-19 pandemic in Germany. PLoS ONE. 2021; 16(3): e0248288. https://doi.org/10.1371/journal. pone.0248288.
4. Liu Z, Magal P, Webb G. Predicting the number of reported and unreported cases for the COVID-19 epidemics in China, South Korea, Italy, France, Germany and United Kingdom, Journal of Theoretical Biology. 2021; 509:110501.
