# Supplementary material for: Mitigating the outbreak of an infectious disease over its life cycle: A diffusion-based approach
Source: PLoS One. 2023 Jan 26;18(1):e0280429. doi: 10.1371/journal.pone.0280429 (PMC9879393; doi:10.1371/journal.pone.0280429)
Supplement: Peer review history

# Author comment: Author response to previous submission

**DOI:** 10.1371/journal.pone.0280429.r001

8 Sep 2022

Attachment Submitted filename: Response to Reviewers.docx Click here for additional data file.

# Editor report: Decision Letter 0

**DOI:** 10.1371/journal.pone.0280429.r002

**Authors:** Chi Ho Yeung

2 Jan 2023

Mitigating the outbreak of an infectious disease over its life cycle: A diffusion-based approach

PONE-D-22-25000

Dear Dr. Li,

We’re pleased to inform you that your manuscript has been judged scientifically suitable for publication and will be formally accepted for publication once it meets all outstanding technical requirements.

Within one week, you’ll receive an e-mail detailing the required amendments. When these have been addressed, you’ll receive a formal acceptance letter and your manuscript will be scheduled for publication.

An invoice for payment will follow shortly after the formal acceptance. To ensure an efficient process, please log into Editorial Manager at http://www.editorialmanager.com/pone/, click the 'Update My Information' link at the top of the page, and double check that your user information is up-to-date. If you have any billing related questions, please contact our Author Billing department directly at authorbilling@plos.org.

If your institution or institutions have a press office, please notify them about your upcoming paper to help maximize its impact. If they’ll be preparing press materials, please inform our press team as soon as possible -- no later than 48 hours after receiving the formal acceptance. Your manuscript will remain under strict press embargo until 2 pm Eastern Time on the date of publication. For more information, please contact onepress@plos.org.

Kind regards,

Chi Ho Yeung

Academic Editor

PLOS ONE

Additional Editor Comments:

The authors have well addressed the concerns from the previous reviewer and the editor, I therefore recommend the manuscript to be accepted for publication, but recommend the authors to have the following revisions on the manuscript before publication:

1) A language proof-reading may be helpful, for instance, whether past tense should be used when we refer to previous works: Siettos and Russo [15] provided a comprehensive review ...

2) It seems that most figures do not have appropriate axis labels. Please state those axis labels in the figures with appropriate units, e.g. Time (days)

Reviewers' comments:

# Editor report: Acceptance letter

**DOI:** 10.1371/journal.pone.0280429.r003

**Authors:** Chi Ho Yeung

13 Jan 2023

PONE-D-22-25000 

Mitigating the outbreak of an infectious disease over its life cycle: A diffusion-based approach 

Dear Dr. Li:

I'm pleased to inform you that your manuscript has been deemed suitable for publication in PLOS ONE. Congratulations! Your manuscript is now with our production department. 

If your institution or institutions have a press office, please let them know about your upcoming paper now to help maximize its impact. If they'll be preparing press materials, please inform our press team within the next 48 hours. Your manuscript will remain under strict press embargo until 2 pm Eastern Time on the date of publication. For more information please contact onepress@plos.org.

If we can help with anything else, please email us at plosone@plos.org. 

Thank you for submitting your work to PLOS ONE and supporting open access. 

Kind regards, 

PLOS ONE Editorial Office Staff

on behalf of

Dr. Chi Ho Yeung 

Academic Editor

PLOS ONE
